# Impact of conditional cash transfer programs on health outcomes in Argentina: a retrospective, observational analysis based on MICS 2019/2020

**DOI:** 10.1016/j.lana.2025.101011

**Published:** 2025-02-13

**Authors:** Zlatko Nikoloski, Maria Elisa Zapata, Elias Mossialos

**Affiliations:** a LSE Health, Department of Health Policy, London School of Economics and Political Science, London, UK; b Centro de Estudios Sobre Nutrición Infantil Dr. Alejandro O'Donnell (CESNI), Ciudad Autónoma de Buenos Aires, Argentina

**Keywords:** Argentina, CCTs, Healthcare, Education, Nutrition

## Abstract

**Background:**

Conditional cash transfers (CCTs) are widely used to combat intergenerational poverty and to invest in human capital. Argentina introduced its own CCT program AUH (*Asignación Universal por Hijo*) in 2009. The aim of this research was to assess the relationship between the AUH program and key indicators: healthcare use, nutritional indicators (among children under five years), and high school enrollment.

**Methods:**

We utilized data from the Multiple Indicators Cluster Survey (MICS) conducted in Argentina between late 2019 and early 2020. Specifically, we employed different matching techniques to estimate the relationship between AUH and healthcare utilization and high school enrolment. Additionally, we assessed the program's importance in improving nutrition outcomes among children under five years.

**Findings:**

Our analysis reveals that the AUH program has not significantly increased healthcare utilization among affiliated children. When accounting for program heterogeneity, the impact of the program was found to be consistent across boys and girls, and across children of different ages, although we found evidence of increased healthcare utilization among adolescents. In addition, there was no statistically significant evidence for a link between program affiliation and reduction in stunting and wasting among children under five years. Furthermore, the program has led to increased high school enrolment among boys, consistent with established findings.

**Interpretation:**

The AUH program demonstrates a limited impact, particularly on health and nutrition outcome indicators. Efforts should be made to improve the program by focusing on cash transfer conditionality and amount, as well as strengthening healthcare infrastructure.

**Funding:**

None.

**Research in context**

**Evidence before this study**

The aim of this research was to assess the relationship between the AUH (*Asignación Universal por Hijo*) program and key indicators: healthcare use, nutritional indicators (among children under five years) and high school enrollment. We used keywords including “conditional cash transfers,” “conditionality,” “education,” “health,” “effectiveness,” “AUH,” and “Argentina” to identify relevant literature in English and Spanish evaluating the effectiveness of the Argentine CCT (Conditional Cash Transfers) program. There is a substantial body of evidence exploring the effects of AUH on outcomes related to human capital. Research papers on the impact of AUH can be categorised into two main groups. The first aims to disentangle the influence of AUH on poverty and labour market outcomes, while the second focuses on analysing the impact of AUH on educational outcomes, and on school enrolment in particular. One study explores both themes. In summary, this body of research consistently demonstrates the positive impact of the AUH program on children's economic well-being, food security, and school attendance. However, there is limited available evidence regarding the effects of the AUH on nutrition outcomes, such as stunting and wasting, among children under five years.

**Added value of this study**

To address these gaps, we utilised data from the Multiple Indicators Cluster Survey (MICS) conducted in Argentina between late 2019 and early 2020. Using propensity score matching and the double robust inverse probability weighted regression adjustment approach, we examined the link between AUH and various outcome variables specifically related to healthcare utilisation, high school enrolment, and nutrition outcomes. Our analysis indicates that the AUH program has not led to a significant increase in healthcare utilisation among enrolled children. An analysis of program heterogeneity revealed a consistent impact across boys and girls and children of different age groups, although there is some indication of increased healthcare utilisation among adolescents. Additionally, there is no statistically significant evidence supporting the role of the program in reducing stunting and wasting among children under five years. Moreover, the program has resulted in increased high school enrolment among boys, which aligns with established findings.

**Implications of all the available evidence**

Overall, we found no evidence for a statistically significant link between AUH program affiliation and health and nutrition-related indicators. Further efforts should be made to improve the program by, inter alia, increasing the amount of the cash transfer as well as strengthening healthcare infrastructure.

## Introduction

Sustainable Development Goal (SDG) 1 centers on livelihood improvement through the reduction of both monetary and non-monetary poverty. One effective approach that has gained momentum since the 1990s is the implementation of conditional cash transfers (CCTs) which aim to break the cycle of intergenerational poverty and promote investments in human capital.^1^ Notably, given the requirement for complementary supply-side inputs, such as school and healthcare centers, CCTs have gained popularity in middle-income countries with pre-existing administrative capacity and accessible health and education services.^2^ The origins of CCTs date back to the late 1990s in Latin America (mostly in Brazil and Mexico), where windfalls from natural resources were used for a pioneering demand-side intervention that combined income transfer with prescribed recipient behavior.^3^, ^4^, ^5^

Overall, CCTs have proven to be effective in achieving SDG 1 objectives by enhancing livelihoods and reducing poverty.^6^ By incorporating conditionalities that address education and health, transfers contribute to the broader goal of breaking the cycle of poverty and facilitating human capital investment.^7^ However, despite the popularity of CCTs, most recent evidence suggests that unconditional cash transfer (UCT) programs could be as effective in reaching desired behavioral changes (e.g., increasing school enrolment^8^ and reducing instances of unwanted teenage pregnancy^9^). Furthermore, some criticism has been directed toward certain CCT programs owing to their potential reinforcement of traditional gender roles.^10^^,^^11^

Extensive research assessing the impact of CCTs on healthcare utilization, including in Colombia,^12^ Peru,^13^ and Brazil^14^ has found that the health conditions attached to CCTs increase healthcare utilization. Furthermore, evidence from Mexico has found positive effects of existing CCTs on nutrition-related outcomes.^15^ In addition, Brazil's *Bolsa Escola* program has been found to lead to increased school enrolment.^16^^,^^17^ Most recently, studies have also attempted to understand the longer-term effects of CCTs,^18^ including indicators such as arrest rates in men and teenage pregnancy in women.^19^

In Argentina, CCTs were first implemented during the mid-2000s in response to the country's economic and political crisis. By 2002, 44.4% of Argentina's population, including a significant share of children, was living in poverty.^20^^,^^21^ In an effort to address this crisis, the Argentine government introduced three concurrent social protection programs: the Heads of Household Program (*Programa Jefes y Jefas de Hogar*), the Family Plan for Social Inclusion (*Plan Familias por la Inclusión Social*), and Training and Employment Insurance (*Seguro de Capacitación y Empleo*).^22^ Although these programs initially demonstrated success, their fragmentation posed significant challenges, particularly the enforcement of conditionality and the administration of the programs as a whole.^23^^,^^24^ Drawing from insights garnered from these programs, a presidential decree was issued to institute the Universal Child Allowance (*Asignación Universal por Hijo*, AUH) program. This innovative step integrated the AUH into the family allowance system, specifically as a non-contributory component.^25^

According to the National Administration for Social Security (ANSES), AUH is the largest social protection program in Argentina, benefiting approximately 4.2 million children below the age of 18.^26^ Eligibility for AUH requires that neither parent is formally employed, that both child and parents have been Argentine residents for a minimum of two years, and that the parents possess national identity cards.^26^ Fulfilling these criteria guarantees access to 80% of the cash transfer. The remaining 20% is only available to the parents of children who fulfil certain additional health and education criteria (e.g., full childhood immunization, regular school attendance).^26^ Compliance is verified annually by the presentation of a signed booklet from relevant health establishments or schools to ANSES.^22^ As of June 2023, the AUH provided a monthly payment of AR$17,093 (US$48.3) per child (full amount of the cash transfer), which, when adjusted for inflation, is comparable to the international poverty line used in upper middle-income countries. The payment amount is adjusted periodically to account for inflation.^22^

In Argentina (as in the rest of the region), CCTs are expected to impact upon the human capital of children by increasing disposable income within the family,^27^, ^28^, ^29^, ^30^, ^31^ increasing female empowerment,^32^ and enforcing the conditionality of the program.^33^^,^^34^ To date, a substantial body of research has documented the impact of AUH on various human capital-related outcomes. One strand of research found a significant impact of AUH on poverty reduction,^35^, ^36^, ^37^, ^38^, ^39^ while another, which focused on education outcomes, found a significant increase in school enrollment among children aged 13 and older,^39^^,^^40^ a reduction in school dropout rates among beneficiary adolescents,^41^ and an overall improvement in school attendance.^42^ Furthermore, some studies have described the AUH or explored its impact on the structure and coverage of Argentina's social protection system,^23^^,^^24^^,^^34^^,^^43^^,^^44^ particularly focusing on the degree of socio-economic homogeneity among beneficiaries,^45^ as well as program eligibility.^46^^,^^47^

Against this background, the objective of this research was to assess the relationship between the AUH program and three key indicators: (i) use of any form of healthcare; (ii) nutritional outcomes (stunting and wasting) among children under five years; and (iii) high school enrolment.

## Methods

### Study design, participants, and procedures

This is a retrospective observational study based on the Multiple Cluster Indicators Survey (MICS) conducted on 15,000 households in Argentina during the 2019/20 period.^48^ MICS is the United Nations’ primary data collection tool used to assess progress toward achieving the SDGs. In Argentina the survey has been conducted twice—in 2012 and, most recently, in 2019/2020. The 19/20 survey sample was carefully designed to yield estimates for numerous indicators related to the well-being of children and women at the national level, as well as within specific residential areas and geographical locations. The sampling approach utilized a multi-stage, stratified cluster sampling method^48^ and, with the use of sampling weights in the analysis, is representative of the urban population across the country. The survey encompassed a diverse range of topics, including health, education, and social protection. Interviewers collected data by administering both household questionnaires and individual questionnaires to women and men aged 15–49 years. Additionally, questionnaires were administered to mothers or caretakers of all children under five years, as well as to randomly selected children aged 5–17 years. For this paper and as per AUH program eligibility criteria, as described above, we limited our focus to eligible children (i.e., children living in households in which both parents are unemployed). We utilized two distinct sub-samples: (i) children aged 0–17, to analyze the utilization of healthcare services and school enrolment and (b) children under five years, to examine child nutrition outcomes (stunting and wasting).

### Statistical analyses

Propensity score matching (PSM) analysis was used to evaluate the relationship between participation in the AUH program and the set of outcome variables. The analysis includes the following outcome variables: (a) use of any type of medical care; (b) severe stunting in children; (c) moderate and severe stunting combined; (d) severe wasting in children; (e) moderate and severe wasting combined; (f) overweight in children; (g) obesity in children (outcome variables (b)-(g) relevant for children under five years); and (h) high school enrolment (in children aged 12–17 years). Enrolment in primary school was not included in the analysis, as primary school enrolment in Argentina is close to universal^20^ (refer to Supplementary Material for additional information on the derivation of outcome variables).

PSM is a well-established technique used to mitigate selection bias in observational data by matching treatment and control units based on observable characteristics.^49^^,^^50^ The treatment group comprised children affiliated with the AUH program, while the control group consisted of children eligible but not affiliated with the AUH program. Our analysis, therefore, compared the outcome variables of children in the treatment group and the control group. We complemented the PSM approach with the doubly robust inverse probability-weighted regression-adjustment (IPWRA) estimator which combines regression analysis to predict treatment and outcome status, making it robust to potential misspecification of either the treatment or the outcome model^51^^,^^52^ (refer to Supplementary Materials for further details of the methodological approach).

As shown in the Supplementary Material, a key assumption underlying propensity score matching methods is that of conditional independence, implying that selection into the treatment group is solely determined by observable characteristics. Moreover, the covariates included in the matching procedure fulfil an important condition for the empirical strategy, as they are derived from the same source and reflect the same environment.^51^^,^^53^ Against this background and to predict the probability of being treated, we utilized the following variables: sex of the child (male or female), age of the child (in years), socio-economic situation of the household (measured by the asset index wealth quintiles), education level of the mother, ethnicity of the household head, and living conditions (indicated by whether the household is in a slum area). The analysis also incorporated regional fixed effects variables (refer to the Supplementary Material for a more comprehensive account of the methodology). The selection of variables for analysis was driven by both existing evidence and availability of data. While the literature has previously included variables capturing the overall health status of mothers as well as distance to healthcare facilities, we did not include these variables as they are not included in the MICS dataset. A simple logit model (refer to Supplementary Material, Table A1 and A2) captures the link between these variables and the probability of being enrolled in AUH, suggesting a significant link between AUH and variables that capture vulnerability to poverty.

The statistical analysis also included conducting the usual PSM diagnostic tests. Furthermore, given the potential problem of simultaneity bias, we conducted a robustness check whereby we excluded living conditions from the list of variables. Finally, we conducted additional disaggregated analysis based on the sex and age of the child. Disaggregated analyses based on the self-reported ethnicity of the household head were not conducted due to the small sample size of respondents who self-described as belonging to an ethnic minority.

All analyses were conducted in Stata 18.5.

### Role of funding source

There was no funding source for this study.

## Results

Before presenting the results of the analytical exercise, we provide an overview of the descriptive statistics. The upper part of Table 1 presents the summary statistics of the independent variables used in the analysis of healthcare utilization and school enrolment. The descriptive statistics for indicators relevant to children under five years are in the Supplementary Material, Table A3.

**Table 1:** Summary of statistics, by AUH affiliation.

	Not treated (%)	Not treated (n)	Treated (%)	Treated (n)
Female	0.47	1709	0.50	3047
Wealth quintiles				
Quintile 1	0.34	1215	0.44	2725
Quintile 2	0.23	818	0.24	1500
Quintile 3	0.21	754	0.19	1152
Quintile 4	0.16	561	0.09	565
Quintile 5	0.07	250	0.03	191
Education of the mother				
Up to secondary	0.60	2164	0.66	4031
Region				
City of BA	0.17	606	0.13	799
The province of BA	0.08	287	0.08	479
Cuyo	0.15	523	0.19	1135
NOA	0.17	600	0.17	1042
NEA	0.22	778	0.24	1483
Patagonia	0.10	373	0.07	454
Pampeana	0.12	431	0.12	741
Household head of ethnic minority	0.07	264	0.07	404
Household living in a slum	0.10	364	0.11	699
Mother's age				
Mother less than 24 years old	0.05	176	0.10	598
Mother over 24 years old	0.95	3422	0.90	5535
Age of the child (in years)	9.66 (5.49)		6.64 (4.79)	
Number of observations	3598	3598	6133	6133

The summary statistics are based on participation in the AUH program. The sample is almost equally divided between girls and boys, and the summaries indicate that 44% of children affiliated with the program (2725 out of 6133) belong to the lowest socio-economic quintile, in line with expectations. In comparison, only 34% of children unaffiliated with the program (1215 out of 3598) belong to the lowest wealth quintile. Meanwhile, only 3% of AUH-affiliated children (191 out of 6133) are in the top wealth quintile, whereas 7% of children unaffiliated with the program (250 out of 3598) belong to this quintile (Table 1).

The two sub-samples, consisting of AUH-affiliated and unaffiliated children, are relatively evenly distributed across Argentina's seven regions (despite some differences), and the two sub-samples exhibit similar average ages for the children. These findings are consistent for an analysis of the sample of children under five years (Supplementary Material, Table A3).

Table 2 presents a concise summary of the analysis outcome variables. The results indicate a slightly higher utilization of healthcare (53% [3268 out of 6133] vs. 45% [1605 out of 3598]; p-value <0.0001) and high school enrolment (95% [1129 out of 1187] vs. 85% [1309 out of 1536], p-value <0.0001) among AUH affiliated children, compared to unaffiliated children. By contrast, an analysis of indicators relevant to children under five years reveals minimal disparities between affiliated and unaffiliated groups (e.g., severe and moderate stunting: 3% [66 out of 2221] vs. 2% [16 out of 732]; p-value: 0.262).

**Table 2:** Summary of outcome variables, by AUH affiliation.

	Not treated (%)	Not treated (n)	Treated (%)	Treated (n)	p-values
Use of healthcare	0.45	1605	0.53	3268	<0.0001
Number of observations	3598		6133		
Enrolment in high school	0.85	1309	0.95	1129	<0.0001
Number of observations	1536		1187		
Stunting (severe)	0.02	16	0.03	66	0.262
Stunting (moderate and severe)	0.09	65	0.09	203	0.832
Wasting (severe)	0.00	3	0.00	11	0.796
Wasting (moderate and severe)	0.02	15	0.02	46	0.970
Overweight	0.10	77	0.13	297	0.059
Obese	0.05	40	0.05	120	0.947
Number of observations	732	732	2221	2221	

Table 3 presents the key findings of the analysis (the full set of results are presented in the Supplementary Materials, Table A4–A11). The matching exercise involved a two-fold approach of propensity score matching followed by inverted probability weighted regression adjustment (IPWRA) score matching. To ensure consistency between the two approaches, the results are presented as average treatment effects (ATE). While the results suggest higher utilization of healthcare among AUH affiliated children (ATE: 0.033; p-value: 0.016; 95% CI [0.006–0.061]), the significance of the result disappears when using the doubly robust IPWRA. Furthermore, the findings also suggest that the AUH program has had no statistically significant link with most of the nutrition-related outcomes, including severe or moderate stunting, overweight, and obesity (Table 3). In contrast, the results of the IPWRA matching exercise indicate that the educational conditionalities of the program have increased high school enrolment (ATE: 0.054; p-value <0.0001; 95% CI [0.033–0.075]).

**Table 3:** Propensity score-matching results, all outcome variables.

	Use of any healthcare	Severe stunting	Moderate and severe stunting	Severe wasting	Severe and moderate wasting	Overweight	Obesity	Secondary education enrolment
psmatch								
ATE	0.033	0.005	0.006	−0.00005	−1.25E-18	6.00E-03	−1.40E-02	0.047
SE	(0.013)	(0.007)	(0.014)	(0.005)	(0.008)	(0.018)	(0.013)	(0.012)
p-value	0.016	0.465	0.673	0.991	1.000	0.703	0.291	<0.0001
95% confidence interval	[0.006–0.061]	[−0.009 to 0.020]	[−0.023 to 0.035]	[−0.010 to 0.010]	[−0.015 to 0.015]	[−0.028 to 0.042]	[−0.041 to 0.012]	[0.022–0.071]
ipwra								
ATE	0.018	0.011	0.006	0.0004	0.0008	0.024	−0.003	0.054
SE	(0.011)	(0.006)	(0.012)	(0.003)	(0.006)	(0.013)	(0.010)	(0.010)
p-value	0.106	0.06	0.59	0.885	0.891	0.076	0.73	<0.0001
95% confidence interval	[−0.003 to 0.041]	[−0.0004 to 0.023]	[−0.017 to 0.030]	[−0.005 to 0.006]	[−0.011 to 0.013]	[−0.002 to 0.051]	[−0.023 to 0.016]	[0.033–0.075]
Number of observations	8660	2875	2875	2847	2847	2847	2847	2186

Note: psmatch, propensity score matching; ipwra, inverse probability-weighted regression-adjustment. P-value is used in order to ascertain statistical significance. ATE, average treatment effect; SE, standard error.

### Sensitivity analysis

Two diagnostic tests were conducted as part of the analysis. The first examined whether the matching procedure satisfied the common support or overlap condition. The common support is the area where the balancing score has positive density for both treatment and comparison units. In other words, no matching could occur in the absence of overlap between the treatment and control groups (for evidence that the common support condition was broadly satisfied, refer to Supplementary Material, Figure A1 for the entire sample and Figure A2 for children under five years). The second diagnostic test assessed whether the matching process successfully reduced bias. After matching, there should be no systematic differences in the distribution of covariates between the treated and control groups. The results of this diagnostic test, presented in Supplementary Material, Tables A12–A19, demonstrate that the standardized differences in the independent variables between the two groups (matched and unmatched) were significantly reduced after matching. This indicates a successful reduction in bias through the matching procedure for each of the above-mentioned outcome variables. The quality of the matches was further ascertained by conducting a postestimation box plot visualization (Supplementary Materials, Graphs B1–B8).

Given the potential problem of simultaneity of impact, we conducted an additional robustness check whereby we excluded living conditions from the list of variables. The results (presented in the Supplementary Materials, Tables A20–A22) further confirm the findings of the main analysis. As an additional robustness check, we conducted logit modelling on the link between AUH and the outcome variables. The results support the findings from the matching exercise (Supplementary Materials, Tables A23–A37).

Furthermore, one of the requirements of a matching exercise is an assessment of the heterogeneity of impact. This robustness check disaggregates the results observed in the main analysis on different sub-groups of the population. In our case, we chose to examine heterogeneity impact based on the sex and age of the child. These findings are presented in Tables 4 and 5, with results for girls and boys, and indicate that affiliation with the AUH led to an increase in school enrolment among boys (ATE: 0.084; p-value < 0.0001; 95% CI [0.056–0.113]).

**Table 4:** Heterogeneity of impact, boys only.

	Use of any healthcare	Severe stunting	Moderate and severe stunting	Severe wasting	Severe and moderate wasting	Overweight	Obesity	Secondary education enrolment
psmatch								
ATE	0.044	0.013	−0.024	−0.0006	0.015	0.007	−0.014	0.09
SE	(0.019)	(0.009)	(0.025)	(0.004)	(0.008)	(0.025)	(0.019)	(0.017)
p-value	0.021	0.146	0.323	0.891	0.059	0.778	0.463	<0.0001
95% confidence interval	[0.006–0.081]	[−0.004 to 0.031]	[−0.073 to 0.024]	[−0.010 to 0.008]	[−0.0005 to 0.030]	[−0.042 to 0.056]	[−0.053 to 0.024]	[0.057–0.124]
ipwra								
ATE	0.01	0.013	−0.002	−0.001	0.007	0.022	−0.003	0.084
SE	(0.015)	(0.008)	(0.017)	(0.005)	(0.008)	(0.018)	(0.014)	(0.014)
p-value	0.521	0.09	0.893	0.814	0.37	0.234	0.806	<0.0001
95% confidence interval	[−0.020 to 0.041]	[−0.002 to 0.029]	[−0.036 to 0.031]	[−0.011 to 0.009]	[−0.009 to 0.024]	[−0.014 to 0.059]	[−0.032 to 0.025]	[0.056–0.113]
Number of observations	4436	1509	1509	1481	1481	1481	1481	1123

Note: psmatch, propensity score matching; ipwra, inverse probability-weighted regression-adjustment. P-value is used to ascertain statistical significance. ATE, average treatment effect; SE, standard error.

**Table 5:** Heterogeneity of impact, girls only.

	Use of any healthcare	Severe stunting	Moderate and severe stunting	Severe wasting	Severe and moderate wasting	Overweight	Obesity	Secondary education enrolment
psmatch								
ATE	0.038	0.007	0.027	0.0029	−0.01	0.023	−0.009	0.021
SE	(0.019)	(0.010)	(0.015)	(0.004)	(0.010)	(0.022)	(0.016)	(0.016)
p-value	0.047	0.481	0.073	0.504	0.336	0.298	0.562	0.205
95% confidence interval	[0.0005–0.076]	[−0.012 to 0.027]	[−0.002 to 0.057]	[−0.005 to 0.011]	[−0.031 to 0.010]	[−0.020 to 0.068]	[−0.042 to 0.023]	[−0.011 to 0.054]
ipwra								
ATE	0.025	0.01	0.022	0.001	−0.005	0.023	−0.003	0.021
SE	(0.016)	(0.008)	(0.016)	(0.003)	(0.009)	(0.020)	(0.014)	(0.015)
p-value	0.13	0.217	0.164	0.626	0.534	0.239	0.794	0.170
95% confidence interval	[−0.007 to 0.057]	[−0.006 to 0.027]	[−0.009 to 0.055]	[−0.005 to 0.008]	[−0.024 to 0.012]	[−0.015 to 0.063]	[−0.032 to 0.024]	[−0.009 to 0.052]
Number of observations	4224	1366	1366	1366	1366	1366	1366	1063

Note: psmatch, propensity score matching; ipwra, inverse probability-weighted regression-adjustment. P-value is used to ascertain statistical significance. ATE, average treatment effect; SE, standard error.

To examine healthcare-seeking behavior across different age groups, we conducted a separate analysis focusing on children in three distinct age ranges: 0–5 years, 6–12 years, and 13–17 years (Table 6). The results broadly indicate the lack of a statistically significant link between program affiliation and healthcare utilization.

**Table 6:** Heterogeneity of impact, by age.

	0–5 years			6–12 years			13–17 years	
	Use of any healthcare			Use of any healthcare			Use of any healthcare	
psmatch	ATE	0.026	psmatch	ATE	0.003	psmatch	ATE	0.072
	SE	(0.023)		SE	(0.023)		SE	(0.026)
	p-value	0.251		p-value	0.873		p-value	0.006
	95% confidence interval	[−0.018 to 0.072]		95% confidence interval	[−0.041 to 0.048]		95% confidence interval	[0.020–0.124]
ipwra	ATE	0.025	ipwra	ATE	0.001	ipwra	ATE	0.055
	SE	(0.018)		SE	(0.018)		SE	(0.022)
	p-value	0.165		p-value	0.951		p-value	0.015
	95% confidence interval	[−0.010 to 0.061]		95% confidence interval	[−0.035 to 0.037]		95% confidence interval	[0.010–0.099]
Number of observations		3691	Number of observations		3189	Number of observations		1780

Note: psmatch, propensity score matching; ipwra, inverse probability-weighted regression-adjustment. p-value is used to ascertain statistical significance. ATE, average treatment effect; SE, standard error.

We also repeated the analysis using bootstrapped standard errors and the results confirm the findings from our principal analysis (Supplementary Materials, Table A38–A40).

## Discussion

This research sought to assess AUH affiliation in relation to its health and education conditionalities. It also examined the program's impact on other indicators, such as stunting and wasting among children under five years. Our findings indicate that, while program affiliation is not associated with increased overall healthcare utilization (except for adolescents), it positively correlates with increased secondary education enrolment, particularly among boys. We also observed that enrollment in the program has not led to a significant reduction in stunting or wasting among children under five years, nor to any significant change in childhood overweight or obesity.

Our primary finding indicates that the link between the AUH program and healthcare utilization is not statistically significant. This contradicts the prevailing evidence in the Latin America region that CCT programs typically increase utilization of healthcare services.^13^^,^^54^^,^^55^ For instance, Mexico's CCT program, PROGRESA, which includes the condition that children undergo annual medical examinations, has been found to result in higher healthcare utilization among children, including older children and adolescents.^56^

The AUH program's lack of observed significance on healthcare utilization prompts several potential explanations. One key challenge that may explain the muted link between the program and healthcare utilization is the indirect linkage between the ANSES enforcement mechanism and the health and education ministries. Indirect mediation through the program booklet^57^ could potentially introduce gaps in ensuring compliance with health and education conditions. In other words, the enforcement mechanism is not directly linked to the ministries of education and health. Moreover, lack of public healthcare infrastructure availability and accessibility have been identified as significant obstacles to increasing healthcare utilization.^58^^,^^59^ Limited resources and capacity within the healthcare system may hinder the ability of beneficiaries to effectively access and receive healthcare services.

Additionally, it is important to consider that the AUH program has undergone significant changes since its inception, increasing the number of conditions, thus potentially influencing healthcare utilization. Initially, the program included two health conditions: regular medical examinations and compliance with the immunization schedule, however, since 2011, a third condition has been added, requiring enrolment in *Plan Nacer*, a government healthcare plan for children under six years. In August 2012, *Plan Nacer* was expanded to encompass children up to 19 years and was renamed *Programa SUMAR*. In general, the addition of more conditions requires a greater number of healthcare visits, which, given the longer waiting times associated with public healthcare facilities, can be a significant time constraint for many families, thus impacting frequency of use. Understanding these modifications is essential for a comprehensive assessment of the program's overall effectiveness in promoting healthcare utilization.

Our findings also suggest a limited link between program affiliation and stunting and wasting in children. Research conducted in other low- and middle-income countries highlights the critical importance of the early years to growth and development. Studies from the Philippines,^60^ Mexico,^61^^,^^62^ Bangladesh,^63^ and Indonesia^64^^,^^65^ have found that the implementation of interventions targeting stunting and wasting during this crucial period can yield positive outcomes. According to the World Development Indicators,^20^ less than 1% of children under five years in Argentina suffer from severe wasting and approximately 2% suffer from wasting. Therefore, the baseline prevalence of these conditions in the population limits the significance and magnitude of the impact observed in our study. Similarly, our results show that there was no association between the program and childhood overweight and obesity. Similar results have been observed in the context of Colombia.^66^ Overall, however, it has been argued that unconditional (rather than conditional) cash transfers have a positive effect on reducing rates of overweight and obesity in children.^67^

Our study reveals that there is a statistically significant association between AUH and high school enrolment, particularly among boys aged 12–17. Given near-universal primary school enrolment in Argentina, our focus was specifically on high school enrolment. This finding is consistent with evidence from Argentina and other countries in the region regarding the educational conditionality of CCT programs which have found educational outcomes to be more pronounced among older children.^39^^,^^40^^,^^68^ Similarly, a study of Brazil's *Bolsa Escola* program indicated that school enrolment increased in response to higher cash transfer amounts.^16^

We did not find evidence that the AUH was linked with the educational outcomes of girls. However, previous research suggests that, although the program is limited in attracting more vulnerable girls and young women currently outside of the education system, it contributes to improving the educational trajectory of girls already enrolled in education, which is evidenced in a reduction in intra-annual dropout rates and an increased likelihood of graduating within the expected time frame.^69^ Furthermore, existing evidence suggests that the AUH has a positive effect on reducing high school dropout rates.^41^

Our research is subject to certain limitations. Firstly, the estimation of the propensity score relies on observable characteristics only and cannot account for unobservable characteristics that may influence both the treatment assignment and the outcomes, potentially introducing bias into our results. However, we have made efforts to include observable characteristics that closely align with the main conditionalities of the AUH program. Secondly, while our analysis includes a treatment variable indicating AUH enrolment, the survey data used does not provide information on duration of affiliation with the program. The length of time enrolled in the AUH could have implications for the program's overall functioning, and the absence of this information may limit our understanding of the program's impact. It is important to note that our sample is limited to urban areas and does not include children from rural areas or small urban localities. This omission may have implications for the outcomes, particularly in relation to healthcare utilization, given possible differences in urban and rural access and utilization patterns. Therefore, the generalizability of our findings to rural populations should be approached with caution. Lastly, enrolment in the program may be impacted by unobservable variables which could simultaneously impact the studied outcomes. The existing coverage evidence suggests potential issues with either incomplete information in the ANSES database or in compliance with conditionality. We mitigated this by controlling for certain variables (mother's education) as well as by the nature of the dataset itself (inclusion of urban families only).

In conclusion, despite the limitations of our study, several important findings emerge regarding the analysis of Argentina's AUH program. Firstly, we observed that the program has not significantly impacted healthcare utilization. In addition, we did not find evidence for the program's impact on reducing stunting and wasting. However, we found that the AUH program has been successful in increasing high school enrolment, particularly among boys.

Several policy implications can be drawn from these findings. Strengthening the enforcement of program conditionalities, preferably under a non-punitive framework, could enhance program impact. This may involve improving coordination between ANSES and the health and education ministries to ensure compliance and improved monitoring of health and education conditions. Additionally, there could be room for flexibility in the conditionalities or a gradual reduction in their strictness. Furthermore, considering the potential benefits and evolving landscape of social protection programs, suggestions have been made to transition the AUH program into a universal cash transfer (UCT) program, gradually shifting toward providing unconditional cash transfers to all eligible households. This might simplify program administration and increase the flexibility and autonomy of beneficiary families.

## Contributors

ZN and EM conceived the study. ZN conducted the analysis with inputs from EM and MEZ. All authors had final responsibility for the submission of the manuscript. ZN and EM accessed and verified the data.

## Data sharing statement

MICS data are available in the public domain: https://mics.unicef.org/

## Declaration of interests

We declare no competing interests.
