# Supplementary material for: Impact of conditional cash transfer programs on health outcomes in Argentina: a retrospective, observational analysis based on MICS 2019/2020
Source: Lancet Reg Health Am. 2025 Feb 13;43:101011. doi: 10.1016/j.lana.2025.101011 (PMC11959375; doi:10.1016/j.lana.2025.101011)
Supplement: Supplementary Material [file mmc1.pdf]

Supplementary materials

Variables used in the model

Outcome variables

Use of any type of care. This variable was based on the question: “Over the last three months, did any type of healthcare consultation take place?” A dummy variable was constructed taking a value of 1 if a healthcare consultation had taken place and 0 if otherwise.

Severe stunting. A dummy variable was constructed taking a value of 1 if the child had a height for age Z (HAZ) score less than 3. A HAZ score is the number of standard deviations of the actual height of a child from the median height of children the same age as determined from the standard sample.

Moderate and severe stunting. A dummy variable was constructed taking a value of 1 if the child had a HAZ score less than 2.

Severe wasting. A dummy variable was constructed taking a value of 1 if the child had a weight for height Z (WHZ) score less than 3. A WHZ score is the number of standard deviations of the actual weight-for-height of a child from the median weight-for-height of children the same age as determined from the standard sample.

Moderate and severe wasting. A dummy variable was constructed taking a value of 1 if the child had a WHZ score less than 2.

Stunting and wasting were assessed according to WHO growth standards.

Overweight. A dummy variable was created taking a value of 1 if the child was overweight and 0 otherwise. As per the WHO definition, overweight is weight-for-height greater than 2 standard deviations above the WHO child growth standards median.

Obese. A dummy variable was created taking a value of 1 if the child was obese and 0 otherwise. As per the WHO definition, obesity is weight-for-height greater than 3 standard deviations above the WHO child growth standards median.

School enrolment. A dummy variable was created based on the question: “Has (name) attended school during the current school year?”

Variables used in the matching process

Sex. A dummy variable was created taking a value of 1 if the child was female and 0 if the child was male.

Age of the child. This continuous variable corresponded to the age of the child. Two ages were used: (i) corresponding to the child’s years (used in the analysis of use of care and school enrolment) and (ii) the child’s age measured in months when analyzing the effect of the program on utilization of diarrhea- and acute respiratory infection (ARI)-related use of healthcare services, and when analyzing nutrition outcomes.

Socio-economic status. Five dummy variables were constructed capturing the quintiles of socio-economic status. The wealth quintiles were created based on the asset index already computed in the survey.

Education of the mother. A dummy variable was constructed taking a value of 1 if the child's mother had completed secondary education or higher and 0 if otherwise.

Ethnicity of the household head. A dummy variable was created taking a value of 1 if the household head is of non-white origin and 0 if otherwise.

Living in a slum. A dummy variable was created if the household was currently living in a slum and 0 if otherwise.

Regional effects. Dummy variables were created for the following regions of the country: (i) metropolitan area of Buenos Aires; (ii) the rest of the province of Buenos Aires; (iii) Cuyo; (iv) Northwest; (v) Northeast; (vi) Pampeana (excluding the rest of the province of Buenos Aires); (vii) Patagonia.

Appendix 3. Propensity score matching

The second robustness check involved propensity score matching (PSM). Several seminal studies have pioneered the use of PSM (e.g., Rosenbaum & Rubin 1983; Dehejia & Wahba 2002; Heckman et al. 1998; Caliendo & Kopeinig 2005; Smith & Todd 2005). Estimation of the average treatment effects on the treatment (ATET) group using matching methods relies on two key assumptions. First, the conditional independence assumption (CIA) implies that selection into the treatment group is solely based on observable characteristics (selection on observables). Second, that the common support or overlap condition is satisfied. Common support is the area where the balancing score has positive density for both treatment and comparison units. No matches can be made to estimate the average treatment effects on the ATET parameter in the absence of overlap between the treatment and non-treatment groups.

When both of these conditions are satisfied, the average treatment impact (ATET) is calculated as follows:

ATET=E(Y1-Y0|D=1)=E(Y1|D=1)-E(Y0|D=1)

While matching, we relied on the usual diagnostic tests, including post matching bias reduction, the likelihood ratio test of the joint significance of all covariates, and the pseudo-R2 from probit of treatment status on covariates after matching on matched sample. After matching, there should be no systematic differences in the distribution of covariates between the treatment and control groups, the pseudo-R2 should be low, and the joint significance of all covariates should be rejected.

More importantly, the correlates included in the matching procedure must satisfy an important condition of the empirical strategy, i.e., they are derived from the same source and from the same environment (Caliendo & Kopeining 2008; Heckman et al. 1999). Thus, the following variables were used to predict the probability of being treated: child's gender (male or female), child's age, socio-economic situation of the household (captured by asset index quintiles), mother's educational attainment, ethnicity of the household head, work status of the household head, and household living conditions (proxied by whether or not the household lives in a slum). Variables capturing regional fixed effects were also used in the analysis.

References:

1. Caliendo, M. and S. Kopeining (2005), "Some practical guidance for the implementation of propensity score matching", IZA Discussion Paper, no. 1588, Bonn, Germany.
2. Dehejia, H.R. and S. Wahba (2002), "Propensity score matching methods for non-experimental causal studies", Review of Economic Statistics 84(1): 151-61.
3. Heckman, J., H. Ichimura, J.Smith and P. Todd (1998), "Characterising Selection Bias Using Experimental Data", Econometrica 66(5): pp.1017-1098.
4. Rosenbaum P.R. and D.B. Rubin (1983), "The central role of the propensity score observational studies for causal effects", Biometrika 70(1): 41-55.
5. Smith, J. and P. Todd (2005), "Does matching overcome LaLonde's critique of non-experimental estimators?", Journal of Econometrics, 125 (1-2): 305-353.

Table A1. Logistic regression – probability of being enrolled in AUH, children aged 0–5 years

auh	Coef.	St.Err.	t-value	p-value	[95% Conf	Interval]	Sig
female	0.865	0.074	-1.69	0.090	0.731	1.023	*
quint1	2.657	0.531	4.89	0.000	1.795	3.931	***
quint2	2.519	0.494	4.72	0.000	1.716	3.699	***
quint3	1.893	0.368	3.28	0.001	1.293	2.770	***
quint4	1.043	0.212	0.21	0.834	0.701	1.554	
o.quint5	1.000	.	.	.	.	.	
uptosecondary	1.367	0.122	3.50	0.000	1.147	1.629	***
region1	0.628	0.103	-2.84	0.005	0.456	0.866	***
region2	1.323	0.266	1.39	0.165	0.891	1.963	
region3	1.614	0.275	2.81	0.005	1.156	2.252	***
region4	0.960	0.160	-0.24	0.808	0.692	1.332	
region5	0.960	0.157	-0.25	0.804	0.697	1.322	
region7	1.063	0.215	0.30	0.764	0.714	1.581	
o.region6	1.000	.	.	.	.	.	
ethnic	0.733	0.127	-1.79	0.074	0.522	1.030	*
o.working	1.000	.	.	.	.	.	
slum	0.920	0.137	-0.56	0.574	0.687	1.231	
mother_less24	1.448	0.184	2.92	0.003	1.130	1.857	***
o.mother_over24	1.000	.	.	.	.	.	
CAGE	1.003	0.003	1.23	0.220	0.998	1.009	
Constant	1.120	0.261	0.49	0.626	0.710	1.767	

Mean dependent var	0.748	SD dependent var	0.434
Pseudo r-squared	0.042	Number of obs	3055.000
Chi-square	142.615	Prob > chi2	0.000
Akaike crit. (AIC)	3337.262	Bayesian crit. (BIC)	3439.679

*** *p<0.01*, ** *p<0.05*, * *p<0.1*

Table A2. Logistic regression – probability of being enrolled in AUH, children aged 0–17 years

auh	Coef.	St.Err.	t-value	p-value	[95% Conf	Interval]	Sig
female	1.094	0.052	1.90	0.058	0.997	1.199	*
quint1	2.672	0.325	8.09	0.000	2.106	3.390	***
quint2	2.324	0.280	6.99	0.000	1.835	2.944	***
quint3	1.856	0.223	5.14	0.000	1.466	2.350	***
quint4	1.232	0.156	1.64	0.101	0.961	1.579	
o.quint5	1.000	.	.	.	.	.	
uptosecondary	1.212	0.062	3.77	0.000	1.097	1.339	***
region1	0.798	0.072	-2.50	0.012	0.668	0.952	**
region2	1.016	0.110	0.15	0.884	0.821	1.257	
region3	1.455	0.131	4.18	0.000	1.220	1.736	***
region4	0.965	0.086	-0.40	0.690	0.810	1.150	
region5	0.960	0.082	-0.48	0.628	0.812	1.134	
region7	0.975	0.105	-0.24	0.813	0.789	1.204	
o.region6	1.000	.	.	.	.	.	
ethnic	0.884	0.084	-1.30	0.193	0.734	1.064	
o.working	1.000	.	.	.	.	.	
slum	0.983	0.075	-0.22	0.822	0.847	1.141	
mother_less24	1.355	0.139	2.96	0.003	1.108	1.656	***
o.mother_over24	1.000	.	.	.	.	.	
schage	0.911	0.004	-18.81	0.000	0.903	0.920	***
Constant	1.562	0.209	3.34	0.001	1.202	2.029	***

Mean dependent var	0.658	SD dependent var	0.474
Pseudo r-squared	0.059	Number of obs	8698.000
Chi-square	607.656	Prob > chi2	0.000
Akaike crit. (AIC)	10543.635	Bayesian crit. (BIC)	10663.839

*** *p<0.01*, ** *p<0.05*, * *p<0.1*

Table A3. Summary of statistics, by AUH affiliation, sub-sample of children 0-5 years

	Not treated (%)	Not treated (n)	Treated (%)	Treated (n)
Female	0.50	400	0.47	1097
Wealth quintiles				0
Quintile 1	0.33	263	0.44	1027
Quintile 2	0.21	170	0.24	572
Quintile 3	0.20	162	0.19	444
Quintile 4	0.17	136	0.09	211
Quintile 5	0.08	63	0.04	88
Education of the mother				0
Up to secondary	0.50	399	0.63	1472
Region		0		0
City of BA	0.20	161	0.13	298
The province of BA	0.07	57	0.09	207
Cuyo	0.14	114	0.20	460
NOA	0.17	132	0.16	371
NEA	0.21	164	0.24	568
Patagonia	0.12	92	0.11	269
Pampeana	0.09	74	0.07	169
Household head of ethnic minority	0.08	60	0.06	140
Household living in a slum	0.09	70	0.10	242
Mother's age				0
Mother less than 24 years old	0.12	96	0.19	441
Motehr over 24 years old	0.88	698	0.81	1901
Age of the child (in months)	30.38 (17.88)		30.86 (16.41)	
Number of observations	794	794	2342	2342

Source: MICS 2019/2020 and authors' estimates

Table A4. Propensity score-matching results, use of any care, children 0–17 years

Propensity score matching

use of care	Coefficient	Robust standard errors	z	P>z	[95% conf. interval]	
ATE						
auh (1 vs 0)	0.033705	0.013982	2.41	0.016	0.006301	0.061109

IPW regression adjustment

use of care	Coefficient	Robust standard errors	z	P>z	[95% conf. interval]	
ATE						
auh (1 vs 0)	0.01857	0.011494	1.62	0.106	-0.00396	0.041097
POmean						
auh						
0	0.495374	0.009576	51.73	0	0.476606	0.514142

Table A5. Propensity score-matching results, secondary education enrollment

Propensity score matching

school enrollment	Coefficient	Robust standard errors	z	P>z	[95% conf. interval]	
ATE						
auh (1 vs 0)	0.047398	0.012457	3.81	0	0.022983	0.071812

IPW regression adjustment

school enrollment	Coefficient	Robust standard errors	z	P>z	[95% conf. interval]	
ATE						
auh (1 vs 0)	0.054433	0.010777	5.05	0	0.033311	0.075556
POmean						
auh						
0	0.900753	0.008569	105.12	0	0.883958	0.917548

Table A6. Propensity score-matching results, severe stunting, children aged 0–5 years

Propensity score matching

severe stunting	Coefficient	Robust standard errors	z	P>z	[95% conf. interval]	
ATE						
auh (1 vs 0)	0.005449	0.007466	0.73	0.465	-0.00918	0.020082

IPW regression adjustment

severe stunting	Coefficient	Robust standard errors	z	P>z	[95% conf. interval]	
ATE						
auh (1 vs 0)	0.011327	0.006025	1.88	0.06	-0.00048	0.023137
POmean						
auh						
0	0.018868	0.004749	3.97	0	0.009561	0.028176

Table A7. Propensity score-matching results, moderate and severe stunting, children aged 0–5 years

Propensity score matching

moderate and severe stunting	Coefficient	Robust standard errors	z	P>z	[95% conf. interval]	
ATE						
auh (1 vs 0)	0.006319	0.014997	0.42	0.673	-0.02307	0.035712

IPW regression adjustment

moderate and severe stunting	Coefficient	Robust standard errors	z	P>z	[95% conf. interval]	
ATE						
auh (1 vs 0)	0.006642	0.012332	0.54	0.59	-0.01753	0.030813
POmean						
auh						
0	0.082177	0.010753	7.64	0	0.061102	0.103252

Table A8. Propensity score-matching results, severe wasting, children aged 0–5 years

Propensity score matching

severe wasting	Coefficient	Robust standard errors	z	P>z	[95% conf. interval]	
ATE						
auh (1 vs 0)	-5.9E-05	0.005157	-0.01	0.991	-0.01017	0.010049

IPW regression adjustment

severe wasting	Coefficient	Robust standard errors	z	P>z	[95% conf. interval]	
ATE						
auh (1 vs 0)	0.000468	0.003228	0.14	0.885	-0.00586	0.006794
POmean						
auh						
0	0.004448	0.002868	1.55	0.121	-0.00117	0.010069

Table A9. Propensity score-matching results, moderate and severe wasting, children aged 0–5 years

Propensity score matching

moderate and severe wasting	Coefficient	Robust standard errors	z	P>z	[95% conf. interval]	
ATE						
auh (1 vs 0)	-1.25E-18	0.008121	0	1	-0.01592	0.015916

IPW regression adjustment

moderate and severe wasting	Coefficient	Robust standard errors	z	P>z	[95% conf. interval]	
ATE						
auh (1 vs 0)	0.000885	0.006459	0.14	0.891	-0.01177	0.013544
POmean						
auh						

0	0.020822	0.005625	3.7	0	0.009798	0.031846

Table A10. Propensity score-matching results, overweight, children aged 0–5 years

Propensity score matching						
overweight	Coefficient	Robust standard errors	z	P>z	[95% conf. interval]	
ATE						
auh (1 vs 0)	0.006879	0.018022	0.38	0.703	-0.02844	0.042202

IPW regression adjustment						
overweight	Coefficient	Robust standard errors	z	P>z	[95% conf. interval]	
ATE						
auh (1 vs 0)	0.024438	0.013768	1.77	0.076	-0.00255	0.051423
POmean						
auh						
0	0.093111	0.011836	7.87	0	0.069912	0.116309

Table A11. Propensity score-matching results, obesity, children aged 0–5 years

Propensity score matching						
obesity	Coefficient	Robust standard errors	z	P>z	[95% conf. interval]	
ATE						
auh (1 vs 0)	-0.01452	0.013737	-1.06	0.291	-0.04144	0.012406

IPW regression adjustment						
obesity	Coefficient	Robust standard errors	z	P>z	[95% conf. interval]	
ATE						
auh (1 vs 0)	-0.00352	0.010181	-0.35	0.73	-0.02347	0.016437
POmean						
auh						

0	0.050133	0.009065	5.53	0	0.032365	0.067901

Table A12. Postestimation balancing properties, use of any care among children aged 0–17 years

	propensity score matching				IPWRA		
	Standardized differences		Variance ratio			Standardized differences	
	Raw	Matched	Raw	Matched		Raw	Matched
female	0.04	-0.01	1.00	1.00	female	0.04	-0.01
quint1	0.20	-0.03	1.09	0.99	quint1	0.20	0.00
quint2	0.04	0.01	1.05	1.02	quint2	0.04	0.00
quint3	-0.05	0.01	0.92	1.02	quint3	-0.05	0.00
quint4	-0.18	0.01	0.64	1.01	quint4	-0.18	0.00
uptosecondary	0.12	-0.01	0.94	1.01	uptosecondary	0.12	0.00
region1	-0.11	-0.02	0.81	0.97	region1	-0.11	0.00
region2	0.00	0.02	1.01	1.05	region2	0.00	0.00
region3	0.10	0.02	1.19	1.04	region3	0.10	0.01
region4	0.00	0.00	1.00	1.01	region4	0.00	-0.01
region5	0.06	-0.01	1.08	0.98	region5	0.06	0.00
region7	-0.08	0.00	0.79	1.00	region7	-0.08	0.00
ethnic	-0.04	0.05	0.89	1.19	ethnic	-0.04	-0.01
slum	0.03	0.05	1.07	1.13	slum	0.03	0.00
mother_less24	0.20	0.01	2.03	1.04	mother_less24	0.20	0.02
schage	-0.46	-0.01	0.83	0.96	schage	-0.46	0.01
Number of observations				8660	Number of observations		

Note: IPWRA: inverse probability-weighted regression-adjustment

Table A13. Postestimation balancing properties, severe stunting, children aged 0–5 years

	propensity score matching			
	Standardized differences		Variance ratio	
	Raw	Matched	Raw	Matched
female	-0.05	0.00	1.00	1.00
quint1	0.20	0.00	1.09	1.00
quint2	0.05	-0.03	1.07	0.96
quint3	-0.03	0.03	0.95	1.05
quint4	-0.22	0.01	0.60	1.02
uptosecondary	0.22	0.01	0.93	0.99
region1	-0.19	0.03	0.70	1.07
region2	0.06	0.07	1.19	1.25
region3	0.17	0.00	1.35	1.00
region4	-0.01	0.00	0.98	1.00
region5	0.05	-0.01	1.07	0.99
region7	-0.08	-0.03	0.79	0.90
ethnic	-0.04	0.02	0.86	1.07
slum	0.04	-0.01	1.11	0.97
mother_less24	0.16	0.02	1.36	1.04
CAGE	0.02	0.02	0.85	0.89
Number of observations			2875	

	IPWRA		
	Standardized differences		
	Raw	Matched	R
female	-0.05	0.00	
quint1	0.20	-0.01	
quint2	0.05	0.01	
quint3	-0.03	0.00	
quint4	-0.22	0.00	
uptosecondary	0.22	-0.01	
region1	-0.19	0.00	
region2	0.06	0.01	
region3	0.17	-0.01	
region4	-0.01	0.01	
region5	0.05	0.01	
region7	-0.08	-0.02	
ethnic	-0.04	-0.02	
slum	0.04	0.00	
mother_less24	0.16	0.02	
CAGE	0.02	0.00	
Number of observations			

Note: IPWRA: inverse probability-weighted regression-adjustment

Table A14. Postestimation balancing properties, severe and moderate stunting among children aged 0–5 years

	propensity score matching					IPWRA		
	Standardized differences		Variance ratio			Standardized differences		
	Raw	Matched	Raw	Matched		Raw	Matched	R
female	-0.05	0.00	1.00	1.00	female	-0.05	0.00	
quint1	0.20	0.00	1.09	1.00	quint1	0.20	-0.01	
quint2	0.05	-0.03	1.07	0.96	quint2	0.05	0.01	
quint3	-0.03	0.03	0.95	1.05	quint3	-0.03	0.00	
quint4	-0.22	0.01	0.60	1.02	quint4	-0.22	0.00	
uptosecondary	0.22	0.01	0.93	0.99	uptosecondary	0.22	-0.01	
region1	-0.19	0.03	0.70	1.07	region1	-0.19	0.00	
region2	0.06	0.07	1.19	1.25	region2	0.06	0.01	
region3	0.17	0.00	1.35	1.00	region3	0.17	-0.01	
region4	-0.01	0.00	0.98	1.00	region4	-0.01	0.01	
region5	0.05	-0.01	1.07	0.99	region5	0.05	0.01	
region7	-0.08	-0.03	0.79	0.90	region7	-0.08	-0.02	
ethnic	-0.04	0.02	0.86	1.07	ethnic	-0.04	-0.02	
slum	0.04	-0.01	1.11	0.97	slum	0.04	0.00	
mother_less24	0.16	0.02	1.36	1.04	mother_less24	0.16	0.02	
CAGE	0.02	0.02	0.85	0.89	CAGE	0.02	0.00	
Number of observations			2875		Number of observations			

Note: IPWRA: inverse probability-weighted regression-adjustment

Table A15. Postestimation balancing properties, severe wasting, children aged 0–5 years

	propensity score matching			
	Standardized differences		Variance ratio	
	Raw	Matched	Raw	Matched
female	-0.05	0.00	1.00	1.00
quint1	0.20	-0.01	1.09	1.00
quint2	0.05	0.01	1.07	1.01
quint3	-0.03	0.00	0.95	1.00
quint4	-0.22	0.00	0.60	1.01
uptosecondary	0.22	-0.01	0.93	1.01
region1	-0.19	0.00	0.70	1.00
region2	0.06	0.01	1.19	1.05
region3	0.17	-0.01	1.35	0.98
region4	-0.01	0.01	0.98	1.01
region5	0.05	0.01	1.07	1.01
region7	-0.08	-0.02	0.79	0.95
ethnic	-0.04	-0.02	0.86	0.93
slum	0.04	0.00	1.11	1.01
mother_less24	0.16	0.02	1.36	1.04
CAGE	0.02	0.00	0.85	0.85
Number of observations			2847	

	IPWRA		
	Standardized differences		
	Raw	Matched	R
female	-0.06	0.00	
quint1	0.20	-0.01	
quint2	0.04	0.01	
quint3	-0.03	0.00	
quint4	-0.23	0.00	
uptosecondary	0.22	-0.01	
region1	-0.17	0.00	
region2	0.05	0.02	
region3	0.15	-0.01	
region4	-0.02	0.00	
region5	0.06	0.01	
region7	-0.08	-0.02	
ethnic	-0.03	-0.02	
slum	0.02	0.01	
mother_less24	0.16	0.02	
CAGE	0.03	0.00	
Number of observations			

Note: IPWRA: inverse probability-weighted regression-adjustment

Table A16. Postestimation balancing properties, severe and moderate stunting, children aged 0–5 years

	propensity score matching					IPWRA		
	Standardized differences		Variance ratio			Standardized differences		
	Raw	Matched	Raw	Matched		Raw	Matched	R
female	-0.06	0.02	1.00	1.00	female	-0.06	0.00	
quint1	0.20	0.01	1.09	1.00	quint1	0.20	-0.01	
quint2	0.04	0.01	1.06	1.01	quint2	0.04	0.01	
quint3	-0.03	-0.01	0.95	0.98	quint3	-0.03	0.00	
quint4	-0.23	-0.01	0.59	0.98	quint4	-0.23	0.00	
uptosecondary	0.22	0.01	0.94	0.99	uptosecondary	0.22	-0.01	
region1	-0.17	0.01	0.72	1.02	region1	-0.17	0.00	
region2	0.05	-0.01	1.18	0.98	region2	0.05	0.02	
region3	0.15	-0.01	1.31	0.98	region3	0.15	-0.01	
region4	-0.02	0.00	0.97	0.99	region4	-0.02	0.00	
region5	0.06	0.00	1.07	1.00	region5	0.06	0.01	
region7	-0.08	0.04	0.77	1.12	region7	-0.08	-0.02	
ethnic	-0.03	0.02	0.89	1.06	ethnic	-0.03	-0.02	
slum	0.02	0.00	1.06	1.00	slum	0.02	0.01	
mother_less24	0.16	0.00	1.35	1.00	mother_less24	0.16	0.02	
CAGE	0.03	0.01	0.85	0.90	CAGE	0.03	0.00	
Number of observations			2847		Number of observations			

Note: IPWRA: inverse probability-weighted regression-adjustment

Table A17. Postestimation balancing properties, overweight, children aged 0–5 years

	propensity score matching			
	Standardized	differences	Variance ratio	
	Raw	Matched	Raw	Matched
female	-0.06	0.02	1.00	1.00
quint1	0.20	0.01	1.09	1.00
quint2	0.04	0.01	1.06	1.01
quint3	-0.03	-0.01	0.95	0.98
quint4	-0.23	-0.01	0.59	0.98
uptosecondary	0.22	0.01	0.94	0.99
region1	-0.17	0.01	0.72	1.02
region2	0.05	-0.01	1.18	0.98
region3	0.15	-0.01	1.31	0.98
region4	-0.02	0.00	0.97	0.99
region5	0.06	0.00	1.07	1.00
region7	-0.08	0.04	0.77	1.12
ethnic	-0.03	0.02	0.89	1.06
slum	0.02	0.00	1.06	1.00
mother_less24	0.16	0.00	1.35	1.00
CAGE	0.03	0.01	0.85	0.90
Number of observations			2847	

	IPWRA			
	Standardized	differences	Variance ra	
	Raw	Matched	Raw	Mat
female	-0.06	0.00	1.00	[illegible]
quint1	0.20	-0.01	1.09	[illegible]
quint2	0.04	0.01	1.06	[illegible]
quint3	-0.03	0.00	0.95	[illegible]
quint4	-0.23	0.00	0.59	[illegible]
uptosecondary	0.22	-0.01	0.94	[illegible]
region1	-0.17	0.00	0.72	[illegible]
region2	0.05	0.02	1.18	[illegible]
region3	0.15	-0.01	1.31	[illegible]
region4	-0.02	0.00	0.97	[illegible]
region5	0.06	0.01	1.07	[illegible]
region7	-0.08	-0.02	0.77	[illegible]
ethnic	-0.03	-0.02	0.89	[illegible]
slum	0.02	0.01	1.06	[illegible]
mother_less24	0.16	0.02	1.35	[illegible]
CAGE	0.03	0.00	0.85	[illegible]
Number of observations			2847	

Note: IPWRA: inverse probability-weighted regression-adjustment

Table A18. Postestimation balancing properties, obesity, children aged 0–5 years

	propensity score matching			
	Standardized	differences	Variance ratio	
	Raw	Matched	Raw	Matched
female	-0.06	0.02	1.00	1.00
quint1	0.20	0.01	1.09	1.00
quint2	0.04	0.01	1.06	1.01
quint3	-0.03	-0.01	0.95	0.98
quint4	-0.23	-0.01	0.59	0.98
uptosecondary	0.22	0.01	0.94	0.99
region1	-0.17	0.01	0.72	1.02
region2	0.05	-0.01	1.18	0.98
region3	0.15	-0.01	1.31	0.98
region4	-0.02	0.00	0.97	0.99
region5	0.06	0.00	1.07	1.00
region7	-0.08	0.04	0.77	1.12
ethnic	-0.03	0.02	0.89	1.06
slum	0.02	0.00	1.06	1.00
mother_less24	0.16	0.00	1.35	1.00
CAGE	0.03	0.01	0.85	0.90
Number of observations			2847	

	IPWRA			
	Standardized	differences	Variance ra	
	Raw	Matched	Raw	Mat
female	-0.06	0.00	1.00	
quint1	0.20	-0.01	1.09	
quint2	0.04	0.01	1.06	
quint3	-0.03	0.00	0.95	
quint4	-0.23	0.00	0.59	
uptosecondary	0.22	-0.01	0.94	
region1	-0.17	0.00	0.72	
region2	0.05	0.02	1.18	
region3	0.15	-0.01	1.31	
region4	-0.02	0.00	0.97	
region5	0.06	0.01	1.07	
region7	-0.08	-0.02	0.77	
ethnic	-0.03	-0.02	0.89	
slum	0.02	0.01	1.06	
mother_less24	0.16	0.02	1.35	
CAGE	0.03	0.00	0.85	
Number of observations			2847	

Note: IPWRA: inverse probability-weighted regression-adjustment

Table A19. Postestimation balancing properties, secondary school enrolment, children aged 12–17 years

	propensity score matching					IPWRA		
	Standardized differences		Variance ratio			Standardized differences		
	Raw	Matched	Raw	Matched		Raw	Matched	R
female	0.04	-0.01	1.00	1.00	female	0.11	0.00	
quint1	0.20	0.00	1.09	1.00	quint1	0.15	0.00	
quint2	0.04	0.00	1.05	1.01	quint2	0.00	0.00	
quint3	-0.05	0.00	0.92	1.00	quint3	-0.02	0.00	
quint4	-0.18	0.00	0.64	0.99	quint4	-0.11	0.00	
uptosecondary	0.12	0.00	0.94	1.00	uptosecondary	0.04	0.00	
region1	-0.11	0.00	0.81	0.99	region1	-0.09	0.00	
region2	0.00	0.00	1.01	1.01	region2	-0.07	0.00	
region3	0.10	0.01	1.19	1.02	region3	0.10	0.00	
region4	0.00	-0.01	1.00	0.99	region4	0.05	0.00	
region5	0.06	0.00	1.08	1.00	region5	0.05	0.00	
region7	-0.08	0.00	0.79	0.99	region7	-0.08	0.00	
ethnic	-0.04	-0.01	0.89	0.98	ethnic	-0.02	0.00	
slum	0.03	0.00	1.07	1.00	slum	0.00	0.00	
mother_less24	0.20	0.02	2.03	1.05	mother_less24	-0.13	-0.04	
schage	-0.46	0.01	0.83	0.90	schage	-0.32	0.00	
Number of observations			2186		Number of observations			

Note: IPWRA: inverse probability-weighted regression-adjustment

Figure A1. Common support for the entire sample of children

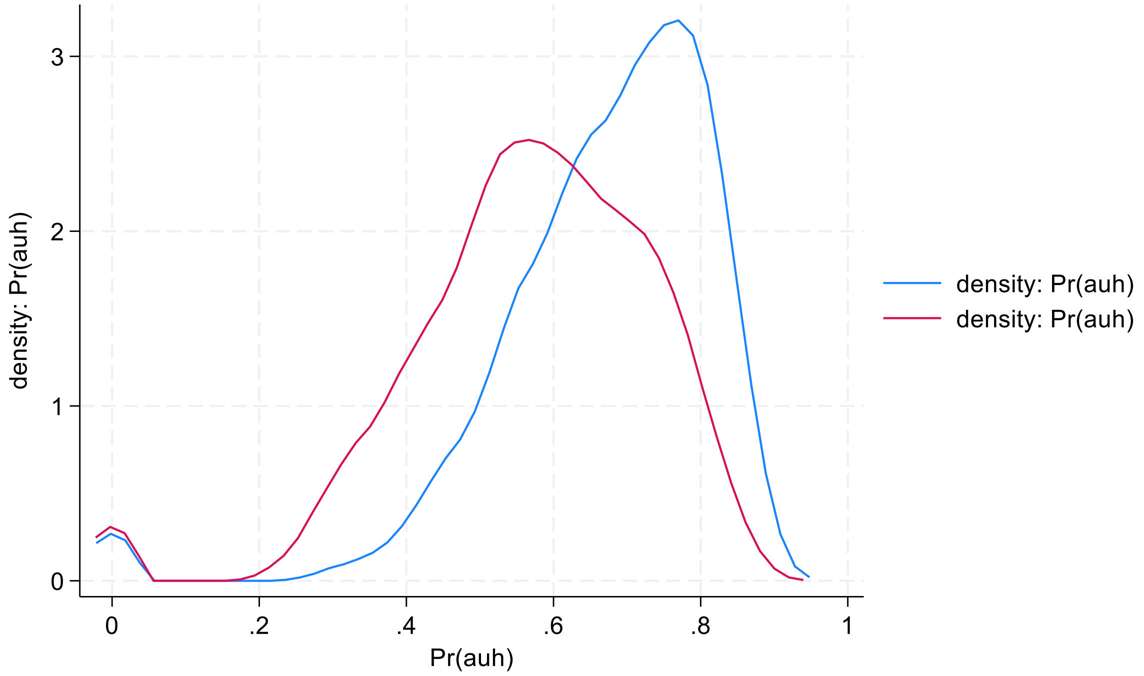

Figure A2. Common support for children aged 0 -5 years

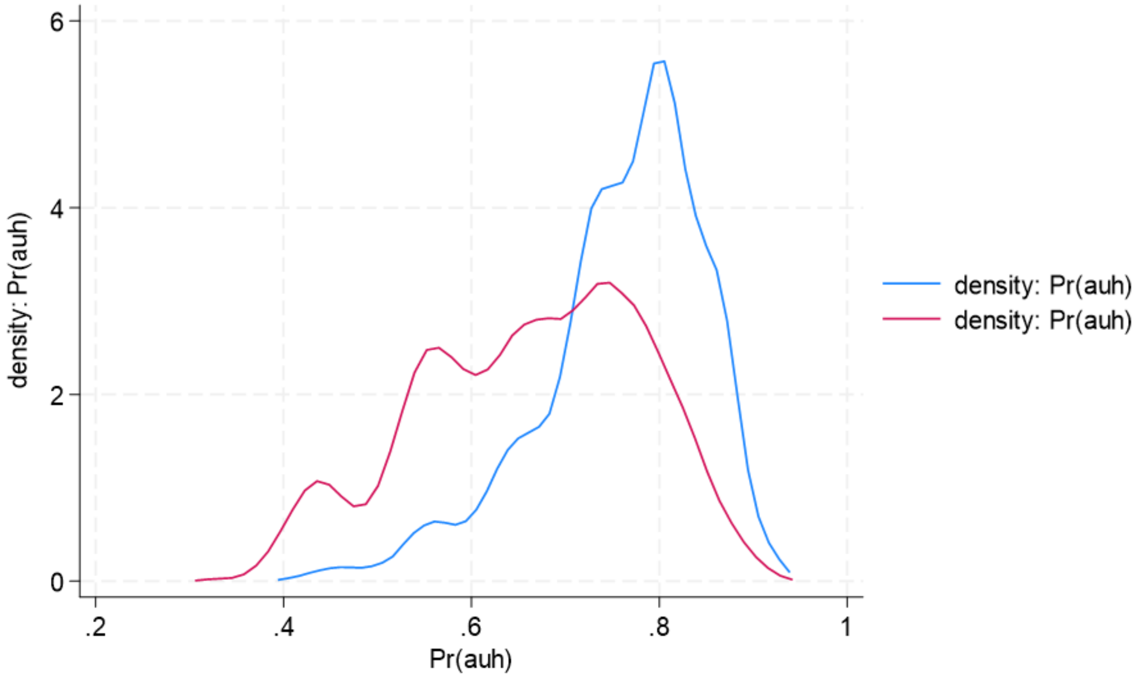

Graph B1. Postestimation box plot, use of any care, children aged 0–17 years

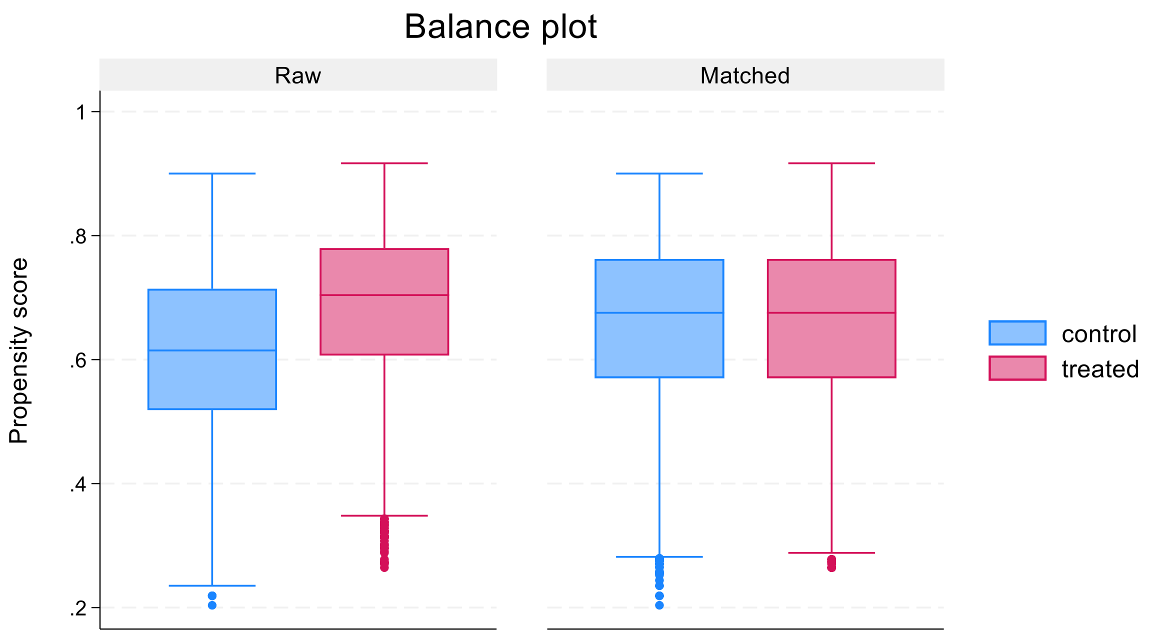

Graph B2. Postestimation box plot, severe stunting, children aged 0–5 years

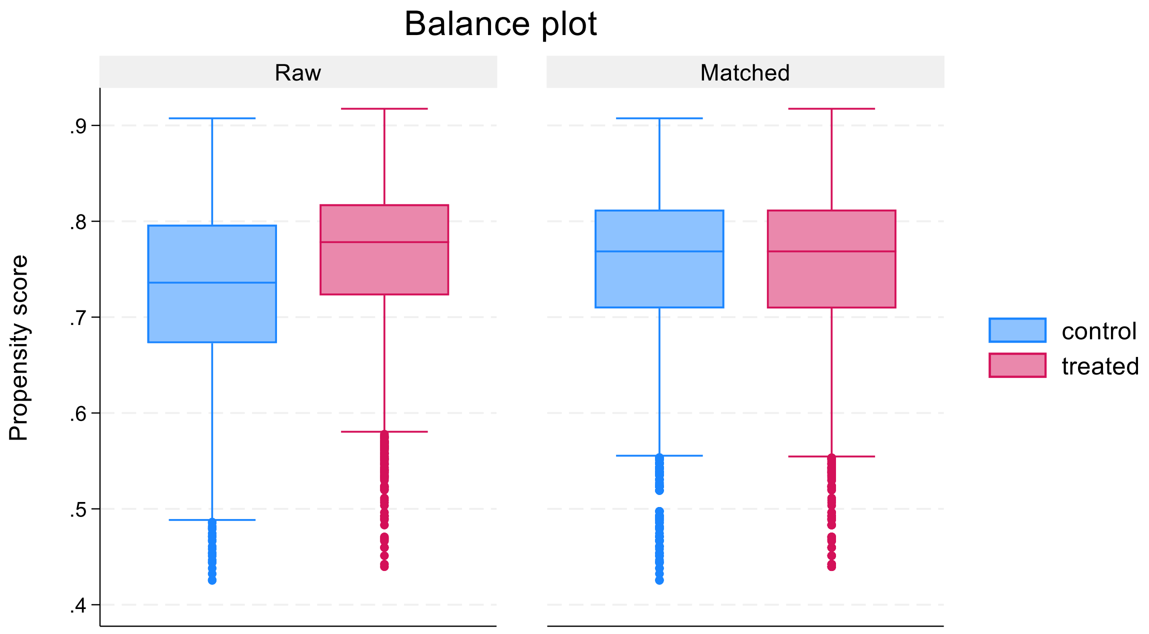

Graph B3. Postestimation box plot, moderate and severe stunting, children aged 0–5 years

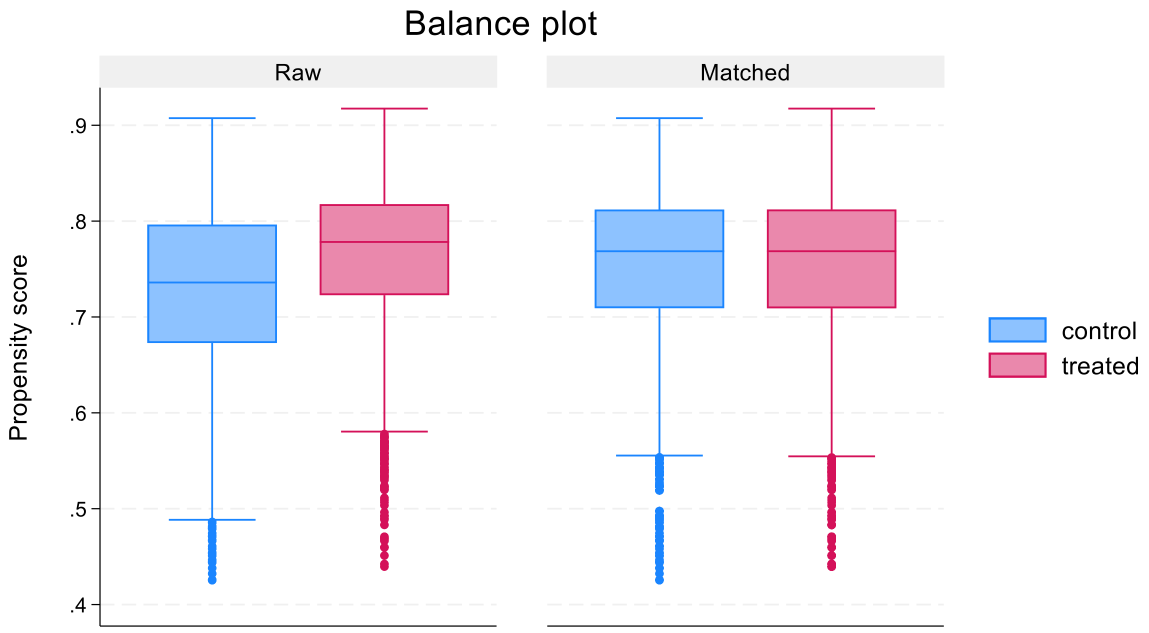

Graph B4. Postestimation box plot, severe wasting, children aged 0–5 years

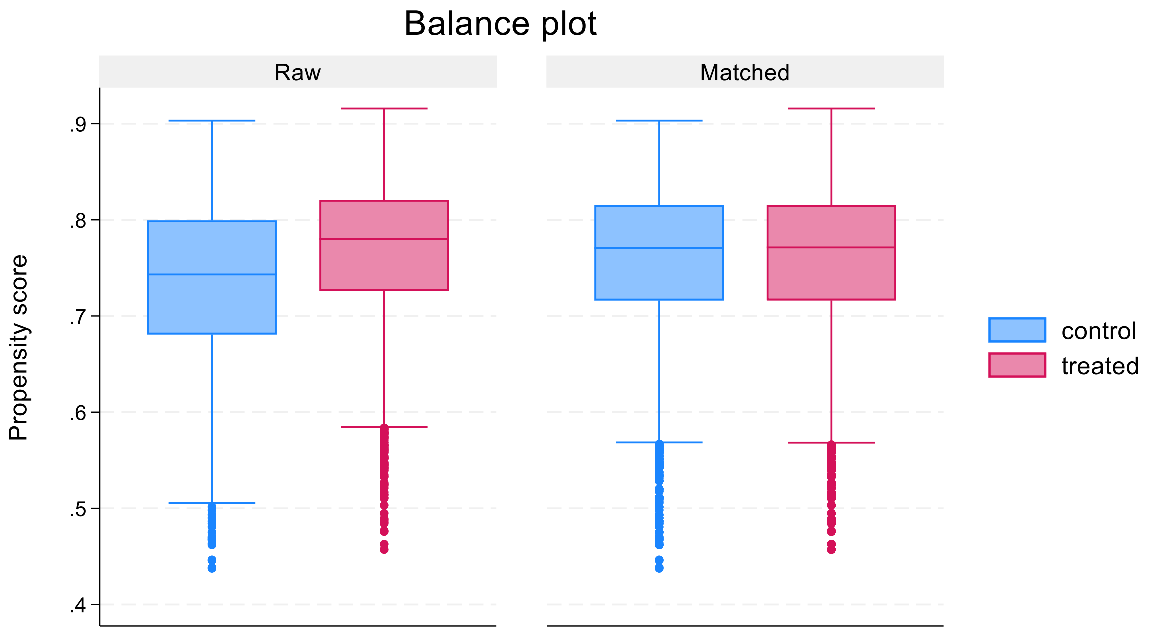

Graph B5. Postestimation box plot, moderate and severe wasting, children aged 0–5 years

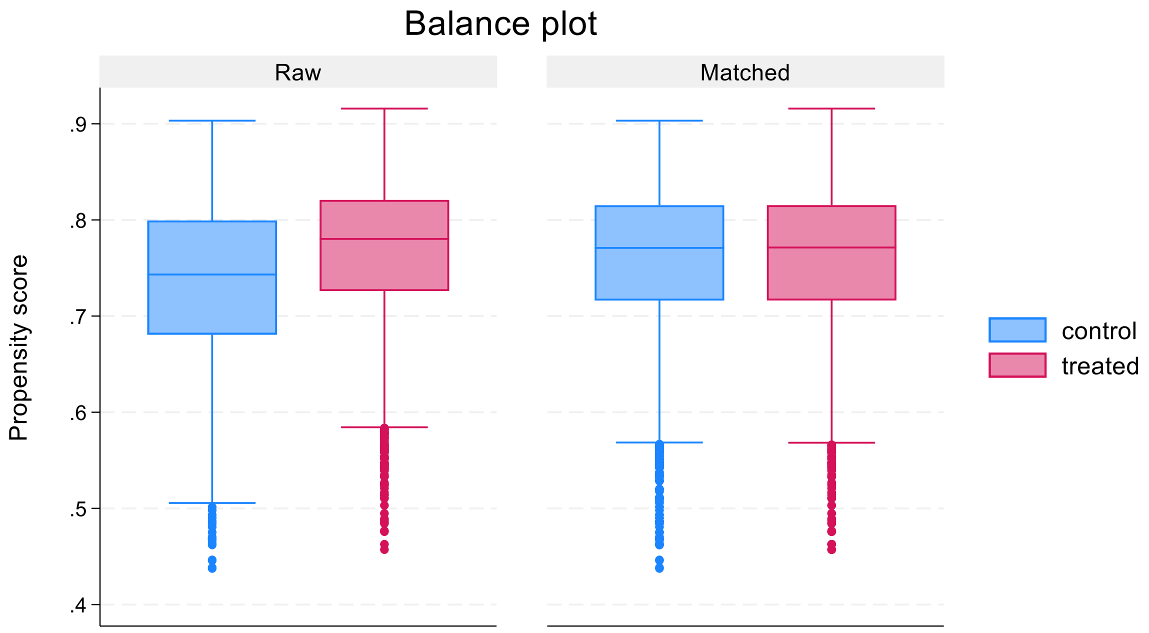

Graph B6. Postestimation box plot, overweight, children aged 0–5 years

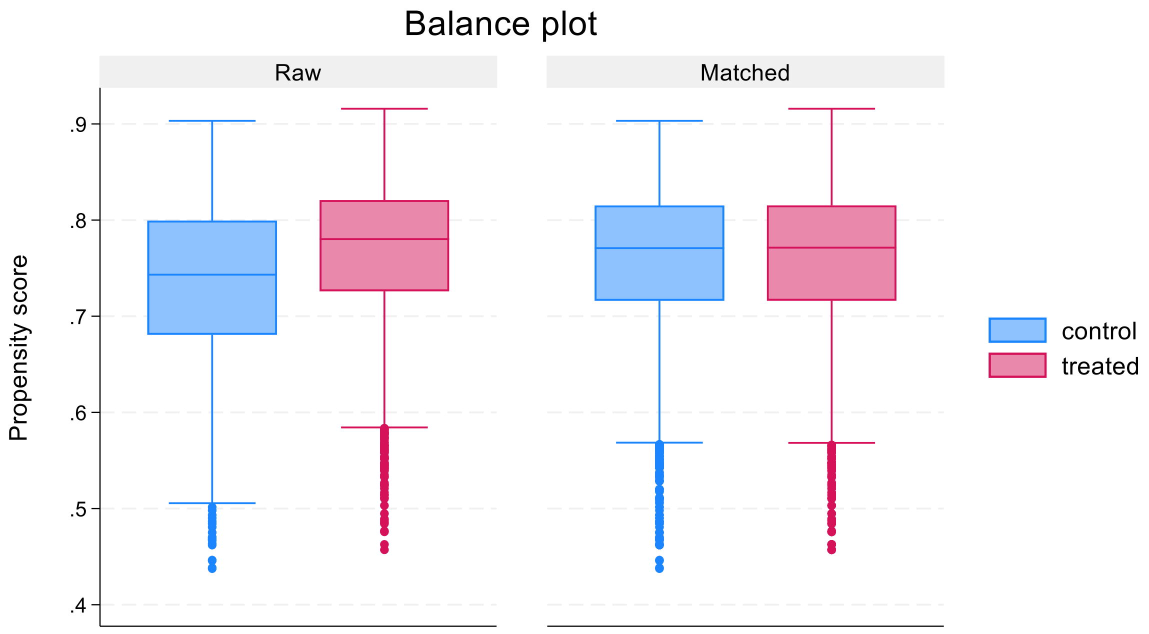

Graph B7. Postestimation box plot, obesity, children aged 0–5 years

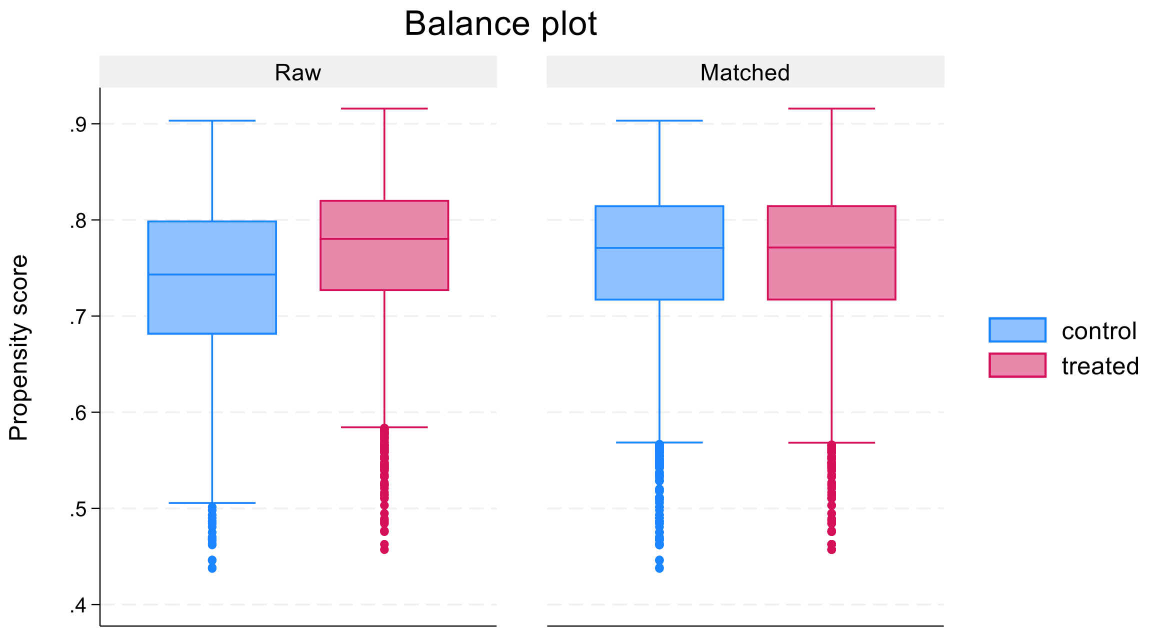

Graph B8. Postestimation box plot, secondary school enrolment

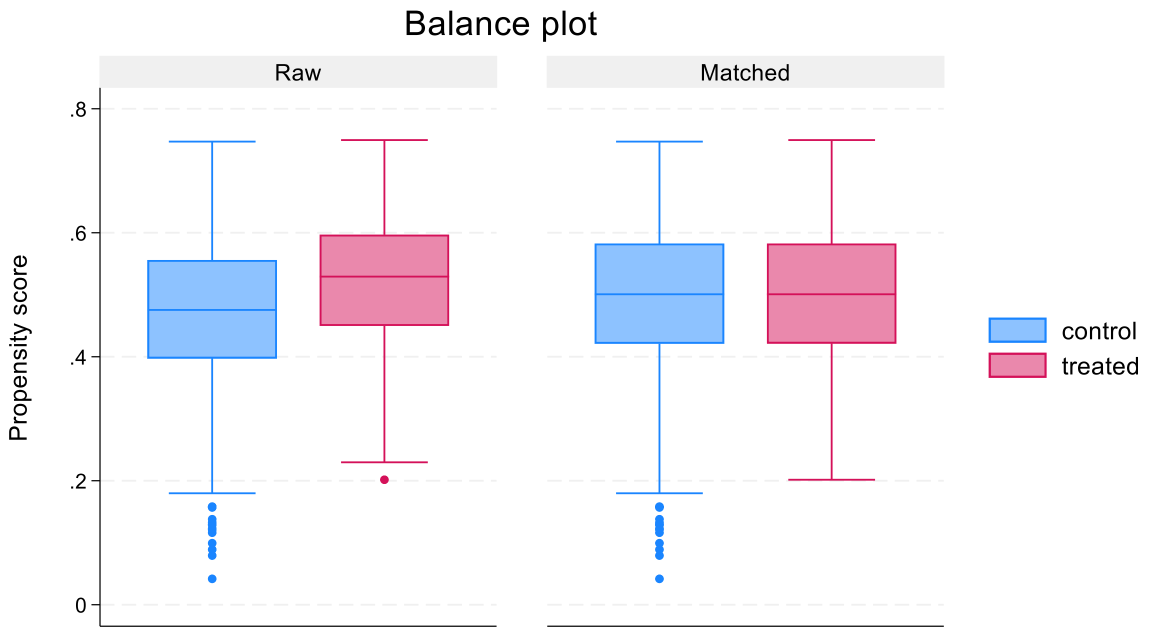

Table A20. Propensity score-matching results, all outcome variables, without the living conditions

		Use of any healthcare	severe stunting	moderate and severe stunting	severe wasting	severe and moderate wasting	overweight	obesity	secondary education enrollment
	ATE	0.02	0.01	0.004	0.00009	-0.003	0.02	-0.01	0.044
	SE	(0.012)	(0.007)	(0.015)	(0.003)	(0.008)	(0.015)	(0.011)	(0.011)
psmatch	sig	n/s	n/s	n/s	n/s	n/s	n/s	n/s	***
	ATE	0.011	0.011	0.007	0.001	0.0008	0.026	-0.001	0.051
	SE	(0.011)	(0.006)	(0.012)	(0.003)	(0.006)	(0.013)	(0.009)	(0.010)
ipwra	sig	n/s	*	n/s	n/s	n/s	n/s	n/s	***
Number of observations		8,660	2,875	2,875	2,847	2,847	2,847	2,847	2,186

Note: psmatch: propensity score matching; ipwra: inverse probability-weighted regression-adjustment.

Source: MICS 2019/2020 and authors' estimates

Table A21a. Heterogeneity of impact, boys only, without living conditions

		Use of any healthcare	severe stunting	moderate and severe stunting	severe wasting	severe and moderate wasting	overweight	obesity	secondary education enrollment
	ATE	0.007	0.012	0.002	0.001	0.009	-0.006	-0.018	0.08
	SE	(0.017)	(0.009)	(0.018)	(0.004)	(0.007)	(0.021)	(0.014)	(0.016)
psmatch	sig	n/s	n/s	n/s	n/s	n/s	n/s	n/s	***
	ATE	0.003	0.012	-0.002	-0.001	0.007	0.021	-0.002	0.08
	SE	(0.015)	(0.008)	(0.017)	(0.005)	(0.008)	(0.018)	(0.014)	(0.014)
ipwra	sig	n/s	n/s	n/s	n/s	n/s	n/s	n/s	***
Number of observations		4,436	1,509	1,509	1,481	1,481	1,481	1,481	1,123

Table A21b. Heterogeneity of impact, girls only, without living conditions

		Use of any healthcare	severe stunting	moderate and severe stunting	severe wasting	severe and moderate wasting	overweight	obesity	secondary education enrollment
	ATE	0.032	0.006	0.016	0.004	-0.012	0.058	0.016	0.011
	SE	(0.018)	(0.008)	(0.019)	(0.003)	(0.012)	(0.023)	(0.017)	(0.016)
psmatch	sig	*	n/s	n/s	n/s	n/s	**	n/s	n/s
	ATE	0.018	0.01	0.021	0.002	-0.006	0.025	-0.004	0.02
ipwra	SE	(0.016)	(0.008)	(0.016)	(0.003)	(0.009)	(0.019)	(0.014)	(0.015)

sig	n/s	n/s	n/s	n/s	n/s	n/s	n/s	n/s
Number of observations	4,224	1,366	1,366	1,366	1,366	1,366	1,366	1,063

Note: psmatch: propensity score matching; ipwra: inverse probability-weighted regression-adjustment. Source: MICS 2019/2020 and authors' estimates

Table A22. Heterogeneity of impact, by age, without living conditions

		0–5 years<br>Use of any healthcare			6–12 years<br>Use of any healthcare			13–17 years<br>Use of any healthcare
	ATE	0.037		ATE	0.0008		ATE	0.046
	SE	(0.022)		SE	(0.021)		SE	(0.024)
psmatch	sig	*	psmatch	sig	n/s	psmatch	sig	*
	ATE	0.022		ATE	-<br>0.0008		ATE	0.049
	SE	(0.018)		SE	(0.018)		SE	(0.022)
ipwra	sig	n/s	ipwra	sig	n/s	ipwra	sig	**
Number of observations		3,691	Number of observations		3,189	Number of observations		1,780

Note: psmatch: propensity score matching; ipwra: inverse probability-weighted regression-adjustment. Source: MICS 2019/2020 and authors' estimates

Table A23. Logistic regression – use of any care, children aged 0–17 years

Use of any care	Coef.	St.Err.	t-value	p-value	[95% Conf	Interval]	Sig
AUH	1.075	.053	1.46	.145	.975	1.184	
female	1.039	.047	0.85	.396	.951	1.135	
quint1	.662	.083	-3.29	.001	.518	.847	***
quint2	.733	.092	-2.48	.013	.574	.937	**
quint3	.835	.104	-1.45	.148	.653	1.066	
quint4	.904	.12	-0.76	.447	.697	1.172	
o	1	.	.	.	.	.	
uptosecondary	.745	.036	-6.04	0	.677	.819	***
region1	.628	.055	-5.27	0	.528	.747	***
region2	.637	.067	-4.30	0	.519	.782	***
region3	1.052	.09	0.59	.555	.89	1.243	
region4	1.166	.1	1.80	.073	.986	1.379	*
region5	.936	.076	-0.81	.415	.799	1.097	
region7	.67	.07	-3.81	0	.546	.823	***
o	1	.	.	.	.	.	
ethnic	1.18	.107	1.83	.068	.988	1.41	*
o	1	.	.	.	.	.	
slum	1.017	.075	0.23	.819	.88	1.174	
mother_less24	1.3	.112	3.05	.002	1.098	1.538	***
o	1	.	.	.	.	.	
schage	.903	.004	-21.04	0	.895	.912	***
Constant	3.607	.51	9.08	0	2.734	4.759	***

Mean dependent var	0.509	SD dependent var	0.500
Pseudo r-squared	0.063	Number of obs	8660
Chi-square	682.465	Prob > chi2	0.000
Akaike crit. (AIC)	11283.491	Bayesian crit. (BIC)	11410.688

**** p<.01, ** p<.05, * p<.1*

Table A24. Logistic regression – probability of being enrolled in secondary school

enrolled	Coef.	St.Err.	t-value	p-value	[95% Conf	Interval]	Sig
AUH	2.41	.464	4.57	0	1.653	3.514	***
female	1.031	.172	0.18	.855	.743	1.43	
quint1	.492	.237	-1.47	.142	.191	1.267	
quint2	.879	.431	-0.26	.792	.336	2.296	
quint3	.696	.339	-0.75	.456	.268	1.806	
quint4	1.72	.976	0.96	.339	.566	5.231	
o	1	.	.	.	.	.	
uptosecondary	.381	.096	-3.85	0	.233	.623	***
region1	1.228	.424	0.59	.552	.624	2.416	
region2	.686	.253	-1.02	.307	.333	1.413	
region3	.948	.295	-0.17	.865	.515	1.746	
region4	.951	.293	-0.16	.87	.519	1.74	
region5	1.356	.406	1.02	.309	.754	2.437	
region7	1.004	.399	0.01	.993	.461	2.186	
o	1	.	.	.	.	.	
ethnic	1.126	.34	0.39	.694	.623	2.036	
o	1	.	.	.	.	.	
slum	1.571	.448	1.58	.114	.898	2.748	
mother_less24	.349	.164	-2.24	.025	.139	.876	**
o	1	.	.	.	.	.	
schage	.57	.038	-8.37	0	.5	.65	***
Constant	112129.28	128842.27	10.12	0	11793.562	1066088	***

Mean dependent var	0.925	SD dependent var	0.263
Pseudo r-squared	0.163	Number of obs	2186
Chi-square	119.090	Prob > chi2	0.000
Akaike crit. (AIC)	1010.522	Bayesian crit. (BIC)	1112.939

**** p<.01, ** p<.05, * p<.1*

Table A25. Logistic regression – use of any care, children aged 0–17 years, boys only

Use of any care	Coef.	St.Err.	t-value	p-value	[95% Conf	Interval]	Sig
AUH	1.049	.073	0.69	.493	.915	1.203	
o	1	.	.	.	.	.	
quint1	.581	.104	-3.03	.002	.408	.826	***
quint2	.682	.122	-2.14	.033	.479	.969	**
quint3	.803	.145	-1.22	.224	.564	1.143	
quint4	.839	.159	-0.93	.354	.579	1.216	
o	1	.	.	.	.	.	
uptosecondary	.767	.052	-3.88	0	.671	.877	***
region1	.629	.077	-3.77	0	.495	.801	***
region2	.585	.087	-3.60	0	.437	.784	***
region3	1.049	.126	0.40	.692	.829	1.326	
region4	1.212	.146	1.59	.111	.957	1.536	
region5	.942	.106	-0.54	.592	.755	1.174	
region7	.652	.096	-2.90	.004	.489	.871	***
o	1	.	.	.	.	.	
ethnic	1.088	.139	0.66	.507	.847	1.398	
o	1	.	.	.	.	.	
slum	1.031	.104	0.31	.76	.846	1.258	
mother_less24	1.606	.192	3.97	0	1.271	2.03	***
o	1	.	.	.	.	.	
schage	.893	.006	-16.38	0	.881	.906	***
Constant	4.207	.835	7.24	0	2.851	6.206	***

Mean dependent var	0.506	SD dependent var	0.500
Pseudo r-squared	0.077	Number of obs	4436
Chi-square	420.871	Prob > chi2	0.000
Akaike crit. (AIC)	5707.928	Bayesian crit. (BIC)	5816.686

**** p<.01, ** p<.05, * p<.1*

Table A26. Logistic regression – probability of being enrolled in secondary school, boys only

enrolled	Coef.	St.Err.	t-value	p-value	[95% Conf	Interval]	Sig
AUH	4.667	1.512	4.75	0	2.473	8.808	***
o	1	.	.	.	.	.	
quint1	.419	.28	-1.30	.194	.113	1.556	
quint2	.681	.458	-0.57	.567	.182	2.543	
quint3	.847	.568	-0.25	.805	.228	3.153	
quint4	3.239	2.732	1.39	.163	.62	16.918	
o	1	.	.	.	.	.	
uptosecondary	.372	.129	-2.85	.004	.189	.734	***
region1	1.606	.77	0.99	.323	.628	4.109	
region2	.669	.328	-0.82	.412	.256	1.748	
region3	1.141	.504	0.30	.765	.48	2.712	
region4	1.336	.6	0.64	.519	.554	3.221	
region5	2.374	1.03	1.99	.046	1.014	5.557	**
region7	.764	.392	-0.52	.6	.279	2.089	
o	1	.	.	.	.	.	
ethnic	1.539	.696	0.95	.341	.634	3.733	
o	1	.	.	.	.	.	
slum	1.862	.812	1.43	.154	.792	4.378	
mother_less24	1.219	1.098	0.22	.826	.209	7.123	
o	1	.	.	.	.	.	
schage	.56	.053	-6.16	0	.466	.673	***
Constant	106785.75	167983.11	7.36	0	4892.16	2330912.7	***

Mean dependent var	0.921	SD dependent var	0.270
Pseudo r-squared	0.234	Number of obs	1123
Chi-square	100.372	Prob > chi2	0.000
Akaike crit. (AIC)	510.256	Bayesian crit. (BIC)	595.660

**** p<.01, ** p<.05, * p<.1*

Table A27. Logistic regression – use of any care, children aged 0–17 years, girls only

Use of any healthcare	Coef.	St.Err.	t-value	p-value	[95% Conf	Interval]	Sig
AUH	1.094	.077	1.27	.203	.953	1.255	
o	1	.	.	.	.	.	
quint1	.762	.133	-1.55	.12	.54	1.074	
quint2	.786	.137	-1.38	.168	.559	1.106	
quint3	.872	.152	-0.79	.43	.62	1.226	
quint4	.975	.182	-0.14	.892	.676	1.405	
o	1	.	.	.	.	.	
uptosecondary	.721	.051	-4.66	0	.629	.828	***
region1	.614	.078	-3.84	0	.478	.788	***
region2	.684	.102	-2.56	.011	.512	.915	**
region3	1.048	.127	0.39	.7	.826	1.33	
region4	1.126	.136	0.98	.327	.888	1.427	
region5	.919	.106	-0.73	.465	.733	1.153	
region7	.679	.102	-2.58	.01	.506	.911	***
o	1	.	.	.	.	.	
ethnic	1.307	.17	2.07	.039	1.014	1.686	**
o	1	.	.	.	.	.	
slum	.995	.106	-0.05	.961	.807	1.227	
mother_less24	1.015	.127	0.12	.906	.794	1.298	
o	1	.	.	.	.	.	
schage	.913	.006	-13.28	0	.901	.926	***
Constant	3.244	.641	5.95	0	2.201	4.779	***

Mean dependent var	0.512	SD dependent var	0.500
Pseudo r-squared	0.051	Number of obs	4224
Chi-square	273.982	Prob > chi2	0.000
Akaike crit. (AIC)	5588.083	Bayesian crit. (BIC)	5696.008

**** p<.01, ** p<.05, * p<.1*

Table A28. Logistic regression – probability of being enrolled in secondary school, girls only

enrolled	Coef.	St.Err.	t-value	p-value	[95% Conf	Interval]	Sig
AUH	1.379	.371	1.20	.232	.814	2.336	
o	1	.	.	.	.	.	
quint1	.655	.489	-0.57	.571	.152	2.829	
quint2	1.209	.929	0.25	.805	.268	5.453	
quint3	.587	.44	-0.71	.477	.135	2.549	
quint4	.965	.803	-0.04	.966	.189	4.932	
o	1	.	.	.	.	.	
uptosecondary	.425	.155	-2.34	.019	.208	.869	**
region1	.98	.521	-0.04	.97	.346	2.778	
region2	.784	.504	-0.38	.705	.222	2.767	
region3	.813	.381	-0.44	.658	.324	2.036	
region4	.654	.303	-0.92	.358	.264	1.62	
region5	.708	.318	-0.77	.441	.294	1.705	
region7	1.559	1.089	0.64	.525	.397	6.132	
o	1	.	.	.	.	.	
ethnic	.748	.308	-0.71	.48	.334	1.675	
o	1	.	.	.	.	.	
slum	1.184	.444	0.45	.652	.568	2.468	
mother_less24	.128	.074	-3.54	0	.041	.399	***
o	1	.	.	.	.	.	
schage	.598	.059	-5.19	0	.492	.726	***
Constant	79941.965	136928.35	6.59	0	2784.818	2294842	***

Mean dependent var	0.929	SD dependent var	0.256
Pseudo r-squared	0.128	Number of obs	1063
Chi-square	57.618	Prob > chi2	0.000
Akaike crit. (AIC)	506.623	Bayesian crit. (BIC)	591.093

**** p<.01, ** p<.05, * p<.1*

Table A29. Logistic regression – use of any care, children aged 0–5 years

use	Coef.	St.Err.	t-value	p-value	[95% Conf	Interval]	Sig
AUH	1.124	.093	1.41	.16	.955	1.323	
female	.952	.068	-0.68	.494	.827	1.096	
quint1	.852	.169	-0.81	.42	.578	1.257	
quint2	.907	.18	-0.49	.624	.616	1.337	
quint3	1.086	.215	0.42	.677	.737	1.6	
quint4	.942	.196	-0.29	.773	.626	1.416	
o	1	.	.	.	.	.	
uptosecondary	.67	.052	-5.14	0	.576	.781	***
region1	.663	.091	-3.00	.003	.507	.868	***
region2	.621	.099	-3.00	.003	.455	.847	***
region3	1.166	.159	1.13	.26	.893	1.522	
region4	1.535	.219	3.00	.003	1.16	2.031	***
region5	1.071	.139	0.53	.599	.831	1.38	
region7	.857	.143	-0.92	.358	.618	1.19	
o	1	.	.	.	.	.	
ethnic	1.133	.17	0.83	.406	.844	1.52	
o	1	.	.	.	.	.	
slum	.904	.107	-0.86	.391	.717	1.139	
mother_less24	1.358	.138	3.01	.003	1.113	1.658	***
o	1	.	.	.	.	.	
schage	.755	.016	-12.85	0	.724	.788	***
Constant	4.673	1.051	6.85	0	3.007	7.263	***

Mean dependent var	0.640	SD dependent var	0.480
Pseudo r-squared	0.058	Number of obs	3691
Chi-square	267.732	Prob > chi2	0.000
Akaike crit. (AIC)	4577.418	Bayesian crit. (BIC)	4689.263

*** *p*<.01, ** *p*<.05, * *p*<.1

Table A30. Logistic regression – use of any care, children aged 6–12 years

use	Coef.	St.Err.	t-value	p-value	[95% Conf	Interval]	Sig
AUH	.993	.077	-0.09	.931	.853	1.157	
female	1.038	.075	0.51	.607	.901	1.196	
quint1	.542	.109	-3.03	.002	.365	.805	***
quint2	.655	.132	-2.11	.035	.442	.971	**
quint3	.769	.156	-1.30	.194	.517	1.143	
quint4	.769	.164	-1.23	.218	.507	1.168	
o	1	.	.	.	.	.	
uptosecondary	.761	.059	-3.51	0	.654	.887	***
region1	.554	.079	-4.17	0	.419	.731	***
region2	.642	.108	-2.65	.008	.463	.892	***
region3	.945	.126	-0.42	.672	.727	1.228	
region4	1.019	.135	0.14	.889	.786	1.321	
region5	.844	.107	-1.34	.181	.658	1.082	
region7	.539	.089	-3.75	0	.39	.744	***
o	1	.	.	.	.	.	
ethnic	1.228	.177	1.43	.153	.926	1.628	
o	1	.	.	.	.	.	
slum	1.133	.133	1.07	.285	.901	1.426	
mother_less24	.928	.204	-0.34	.734	.603	1.428	
o	1	.	.	.	.	.	
schage	.945	.018	-3.02	.003	.912	.981	***
Constant	2.872	.795	3.81	0	1.669	4.941	***

Mean dependent var	0.444	SD dependent var	0.497
Pseudo r-squared	0.020	Number of obs	3189
Chi-square	82.356	Prob > chi2	0.000
Akaike crit. (AIC)	4331.204	Bayesian crit. (BIC)	4440.419

**** p<.01, ** p<.05, * p<.1*

Table A31. Logistic regression – use of any care, children aged 13–17 years

use	Coef.	St.Err.	t-value	p-value	[95% Conf	Interval]	Sig
AUH	1.262	.13	2.27	.023	1.032	1.544	**
female	1.303	.132	2.62	.009	1.069	1.589	***
quint1	.673	.171	-1.55	.12	.409	1.109	
quint2	.622	.157	-1.88	.06	.379	1.02	*
quint3	.627	.158	-1.85	.064	.382	1.028	*
quint4	1.084	.284	0.31	.758	.649	1.81	
o	1	.	.	.	.	.	
uptosecondary	.795	.092	-1.98	.047	.633	.997	**
region1	.714	.14	-1.71	.087	.486	1.05	*
region2	.668	.169	-1.60	.11	.408	1.096	
region3	1.039	.192	0.21	.835	.724	1.492	
region4	.931	.174	-0.39	.7	.646	1.341	
region5	.818	.146	-1.12	.262	.577	1.161	
region7	.698	.168	-1.49	.136	.435	1.12	
o	1	.	.	.	.	.	
ethnic	1.136	.22	0.66	.51	.778	1.659	
o	1	.	.	.	.	.	
slum	1.112	.174	0.68	.498	.818	1.511	
mother_less24	.694	.413	-0.61	.539	.216	2.231	
o	1	.	.	.	.	.	
schage	.992	.037	-0.21	.833	.923	1.067	
Constant	.909	.552	-0.16	.875	.277	2.989	

Mean dependent var	0.352	SD dependent var	0.478
Pseudo r-squared	0.019	Number of obs	1780
Chi-square	40.674	Prob > chi2	0.001
Akaike crit. (AIC)	2302.801	Bayesian crit. (BIC)	2401.519

*** *p*<.01, ** *p*<.05, * *p*<.1

Table A32. Logistic regression – probability of severe stunting, children aged 0–5 years

stunting_severe	Coef.	St.Err.	t-value	p-value	[95% Conf	Interval]	Sig
AUH	1.523	.435	1.47	.141	.87	2.665	
female	1.068	.244	0.29	.775	.682	1.672	
quint1	2.066	1.537	0.98	.329	.481	8.88	
quint2	1.839	1.382	0.81	.417	.422	8.022	
quint3	2.023	1.525	0.93	.35	.462	8.865	
quint4	2.644	2.072	1.24	.215	.569	12.281	
o	1	.	.	.	.	.	
uptosecondary	.547	.137	-2.42	.016	.335	.892	**
region1	.762	.352	-0.59	.556	.308	1.883	
region2	.675	.375	-0.71	.479	.227	2.003	
region3	.504	.237	-1.46	.145	.2	1.267	
region4	1.302	.523	0.66	.51	.593	2.859	
region5	.473	.211	-1.68	.093	.197	1.133	*
region7	1.921	.861	1.46	.145	.798	4.622	
o	1	.	.	.	.	.	
ethnic	.509	.312	-1.10	.27	.153	1.69	
o	1	.	.	.	.	.	
slum	2.014	.691	2.04	.041	1.029	3.945	**
mother_less24	1.112	.333	0.35	.724	.618	2	
o	1	.	.	.	.	.	
CAGE	.979	.007	-3.13	.002	.966	.992	***
Constant	.028	.025	-4.11	0	.005	.155	***

Mean dependent var	0.028	SD dependent var	0.165
Pseudo r-squared	0.054	Number of obs	2875
Chi-square	37.918	Prob > chi2	0.003
Akaike crit. (AIC)	727.122	Bayesian crit. (BIC)	834.470

**** p<.01, ** p<.05, * p<.1*

Table A33. Logistic regression – probability of moderate and severe stunting, children aged 0–5 years

stunting_moderate _~e	Coef.	St.Err.	t-value	p-value	[95% Conf	Interval]	Sig
AUH	1.05	.162	0.32	.751	.776	1.422	
female	.898	.12	-0.81	.419	.691	1.166	
quint1	1.524	.583	1.10	.27	.721	3.224	
quint2	1.181	.455	0.43	.667	.555	2.514	
quint3	1.002	.391	0.01	.995	.466	2.154	
quint4	1.206	.487	0.46	.642	.547	2.662	
o	1	.	.	.	.	.	
uptosecondary	.991	.145	-0.06	.951	.744	1.321	
region1	.741	.2	-1.11	.266	.437	1.256	
region2	.663	.214	-1.27	.204	.352	1.249	
region3	.586	.157	-1.99	.046	.347	.991	**
region4	1.36	.326	1.29	.199	.851	2.175	
region5	.628	.153	-1.91	.056	.39	1.012	*
region7	1.271	.369	0.82	.41	.719	2.246	
o	1	.	.	.	.	.	
ethnic	.369	.144	-2.56	.01	.172	.792	**
o	1	.	.	.	.	.	
slum	1.359	.277	1.51	.132	.911	2.026	
mother_less24	1.069	.186	0.38	.702	.759	1.504	
o	1	.	.	.	.	.	
CAGE	.991	.004	-2.26	.024	.983	.999	**
Constant	.121	.054	-4.76	0	.051	.289	***

Mean dependent var	0.089	SD dependent var	0.285
Pseudo r-squared	0.027	Number of obs	2875
Chi-square	52.487	Prob > chi2	0.000
Akaike crit. (AIC)	1715.461	Bayesian crit. (BIC)	1822.809

**** p<.01, ** p<.05, * p<.1*

Table A34. Logistic regression – probability of severe wasting, children 0–5 years

wasting_severe	Coef.	St.Err.	t-value	p-value	[95% Conf	Interval]	Sig
AUH	1.11	.714	0.16	.871	.314	3.918	
female	.86	.481	-0.27	.787	.287	2.571	
quint1	.395	.473	-0.78	.438	.038	4.121	
quint2	.66	.737	-0.37	.71	.074	5.894	
quint3	.408	.499	-0.73	.463	.037	4.482	
o	1	.	.	.	.	.	
o	1	.	.	.	.	.	
uptosecondary	.914	.523	-0.16	.875	.298	2.807	
region1	.54	.514	-0.65	.518	.083	3.495	
region2	.768	.691	-0.29	.77	.132	4.476	
region3	.216	.245	-1.35	.177	.023	1.998	
region4	.253	.305	-1.14	.255	.024	2.7	
region5	.856	.628	-0.21	.832	.203	3.609	
o	1	.	.	.	.	.	
o	1	.	.	.	.	.	
ethnic	2.685	2.083	1.27	.203	.587	12.288	
o	1	.	.	.	.	.	
o	1	.	.	.	.	.	
mother_less24	1.8	1.016	1.04	.298	.595	5.443	
o	1	.	.	.	.	.	
CAGE	1	.014	-0.01	.995	.973	1.028	
Constant	.019	.027	-2.80	.005	.001	.305	***

Mean dependent var	0.007	SD dependent var	0.081
Pseudo r-squared	0.045	Number of obs	2132
Chi-square	15.976	Prob > chi2	0.315
Akaike crit. (AIC)	191.075	Bayesian crit. (BIC)	276.047

**** p<.01, ** p<.05, * p<.1*

Table A35. Logistic regression- probability of moderate and severe wasting, children aged 0–5 years

wasting_moderate _s~e	Coef.	St.Err.	t-value	p-value	[95% Conf	Interval]	Sig
AUH	1.062	.331	0.19	.846	.577	1.957	
female	1.007	.267	0.03	.978	.599	1.695	
quint1	.354	.218	-1.69	.092	.106	1.183	*
quint2	.633	.386	-0.75	.453	.192	2.089	
quint3	.752	.452	-0.47	.635	.232	2.441	
quint4	.64	.423	-0.68	.5	.175	2.339	
o	1	.	.	.	.	.	
uptosecondary	1.091	.305	0.31	.755	.631	1.889	
region1	.354	.161	-2.29	.022	.146	.862	**
region2	.297	.169	-2.14	.033	.098	.904	**
region3	.237	.104	-3.27	.001	.1	.561	***
region4	.277	.128	-2.77	.006	.112	.686	***
region5	.531	.194	-1.73	.083	.259	1.086	*
region7	.068	.072	-2.53	.012	.008	.547	**
o	1	.	.	.	.	.	
ethnic	.56	.414	-0.78	.432	.131	2.383	
o	1	.	.	.	.	.	
slum	.878	.434	-0.26	.792	.333	2.313	
mother_less24	1.042	.367	0.12	.906	.523	2.078	
o	1	.	.	.	.	.	
CAGE	.995	.007	-0.72	.474	.981	1.009	
Constant	.11	.071	-3.42	.001	.031	.391	***

Mean dependent var	0.021	SD dependent var	0.145
Pseudo r-squared	0.044	Number of obs	2847
Chi-square	32.934	Prob > chi2	0.011
Akaike crit. (AIC)	599.577	Bayesian crit. (BIC)	706.750

**** p<.01, ** p<.05, * p<.1*

Table A36. Logistic regression – probability of being overweight, children aged 0–5 years

overweight	Coef.	St.Err.	t-value	p-value	[95% Conf	Interval]	Sig
AUH	1.443	.229	2.31	.021	1.057	1.969	**
female	1.378	.171	2.59	.01	1.081	1.756	***
quint1	1.117	.379	0.33	.744	.574	2.174	
quint2	1.277	.431	0.72	.469	.659	2.473	
quint3	1.45	.489	1.10	.271	.749	2.808	
quint4	1.296	.464	0.72	.469	.642	2.616	
o	1	.	.	.	.	.	
uptosecondary	.782	.101	-1.92	.055	.608	1.006	*
region1	1.277	.314	1.00	.32	.789	2.066	
region2	1.371	.373	1.16	.247	.804	2.338	
region3	.655	.169	-1.64	.102	.394	1.087	
region4	1.378	.324	1.36	.173	.869	2.185	
region5	.914	.218	-0.38	.706	.572	1.459	
region7	1.965	.533	2.49	.013	1.155	3.344	**
o	1	.	.	.	.	.	
ethnic	.489	.156	-2.24	.025	.261	.915	**
o	1	.	.	.	.	.	
slum	.7	.172	-1.45	.147	.432	1.133	
mother_less24	1.091	.182	0.53	.599	.787	1.513	
o	1	.	.	.	.	.	
CAGE	.998	.004	-0.56	.575	.99	1.006	
Constant	.073	.03	-6.49	0	.033	.161	***

Mean dependent var	0.108	SD dependent var	0.311
Pseudo r-squared	0.028	Number of obs	2847
Chi-square	55.505	Prob > chi2	0.000
Akaike crit. (AIC)	1931.748	Bayesian crit. (BIC)	2038.921

*** *p*<.01, ** *p*<.05, * *p*<.1

Table A37. Logistic regression – probability of being obese, children aged 0–5 years

obesity	Coef.	St.Err.	t-value	p-value	[95% Conf	Interval]	Sig
AUH	1.032	.227	0.14	.888	.67	1.589	
female	1.318	.242	1.50	.133	.919	1.889	
quint1	1.166	.575	0.31	.755	.444	3.064	
quint2	1.4	.69	0.68	.494	.533	3.677	
quint3	1.548	.771	0.88	.38	.583	4.11	
quint4	1.362	.728	0.58	.563	.477	3.885	
o	1	.	.	.	.	.	
uptosecondary	.97	.183	-0.16	.87	.67	1.403	
region1	1.738	.649	1.48	.139	.836	3.614	
region2	2.025	.815	1.75	.079	.921	4.455	*
region3	.706	.291	-0.84	.398	.314	1.584	
region4	1.504	.564	1.09	.276	.721	3.136	
region5	.858	.33	-0.40	.691	.404	1.825	
region7	2.569	1.011	2.40	.017	1.188	5.557	**
o	1	.	.	.	.	.	
ethnic	.301	.175	-2.07	.038	.097	.938	**
o	1	.	.	.	.	.	
slum	.914	.311	-0.27	.791	.469	1.781	
mother_less24	.875	.228	-0.51	.61	.525	1.46	
o	1	.	.	.	.	.	
CAGE	.998	.006	-0.40	.689	.986	1.01	
Constant	.028	.017	-5.79	0	.009	.095	***

Mean dependent var	0.046	SD dependent var	0.209
Pseudo r-squared	0.032	Number of obs	2847
Chi-square	39.933	Prob > chi2	0.001
Akaike crit. (AIC)	1059.118	Bayesian crit. (BIC)	1166.291

**** p<.01, ** p<.05, * p<.1*

Table A38. Propensity score-matching results, all outcome variables, bootstrapped standard errors

		Use of any healthcare	severe stunting	moderate and severe stunting	severe wasting	severe and moderate wasting	overweight	obesity	secondary education enrollment
	ATE	0.018	0.011	0.006	0.0004	0.0008	0.024	-0.003	0.054
	SE	(0.012)	(0.005)	(0.010)	(0.002)	(0.007)	(0.013)	(0.010)	(0.010)
ipwra	sig	n/s	*	n/s	n/s	n/s	n/s	n/s	***
Number of observations		8,660	2,875	2,875	2,847	2,847	2,847	2,847	2,186

Note: psmatch: propensity score matching; ipwra: inverse probability-weighted regression-adjustment.

Source: MICS 2019/2020 and authors' estimates

Table A39a. Heterogeneity of impact, boys only, bootstrapped standard errors

		Use of any healthcare	severe stunting	moderate and severe stunting	severe wasting	severe and moderate wasting	overweight	obesity	secondary education enrollment
	ATE	0.01	0.013	-0.002	-0.001	0.007	0.022	-0.003	0.084
	SE	(0.014)	(0.010)	(0.016)	(0.006)	(0.008)	(0.016)	(0.015)	(0.012)
ipwra	sig	n/s	n/s	n/s	n/s	n/s	n/s	n/s	***
Number of observations		4,436	1,509	1,509	1,481	1,481	1,481	1,481	1,123

Table A39b. Heterogeneity of impact, girls only, bootstrapped standard errors

		Use of any healthcare	severe stunting	moderate and severe stunting	severe wasting	severe and moderate wasting	overweight	obesity	secondary education enrollment
	ATE	0.025	0.01	0.022	0.001	-0.005	0.023	-0.003	0.021
	SE	(0.017)	(0.009)	(0.018)	(0.003)	(0.009)	(0.019)	(0.013)	(0.014)
ipwra	sig	n/s	n/s	n/s	n/s	n/s	n/s	n/s	n/s
Number of observations		4,224	1,366	1,366	1,366	1,366	1,366	1,366	1,063

Note: psmatch: propensity score matching; ipwra: inverse probability-weighted regression-adjustment. Source: MICS 2019/2020 and authors' estimates

Table A40. Heterogeneity of impact, by age, bootstrapped standard errors

		0–5 years			6–12 years			13–17 years
		Use of any healthcare			Use of any healthcare			Use of any healthcare
	ATE	0.025		ATE	0.001		ATE	0.055
	SE	(0.017)		SE	(0.015)		SE	(0.021)
ipwra	sig	n/s	ipwra	sig	n/s	ipwra	sig	**
Number of observations		3,691	Number of observations		3,189	Number of observations		1,780

Note: psmatch: propensity score matching; ipwra: inverse probability-weighted regression-adjustment. Source: MICS 2019/2020 and authors' estimates
